# Peptides Derived from α-Tubulin Induce Functional T Regulatory Cells

**DOI:** 10.3390/ijms26178356

**Published:** 2025-08-28

**Authors:** Tara Fiyouzi, Jose L. Subiza, Esther M. Lafuente, Pedro A. Reche

**Affiliations:** 1 Department of Immunology, Ophthalmology and ENT, Faculty of Medicine, Complutense University of Madrid, Pza Ramon y Cajal s/n, 28040 Madrid, Spain or tarafiyo@ucm.es (T.F.); melafuente@med.ucm.es (E.M.L.); 2 Inmunotek SL, Calle Punto Mobi, 5, 28805 Alcalá de Henares, Spain; jlsubiza@inmunotek.com

**Keywords:** regulatory T cell, epitope, peptide, α-tubulin, immunosuppression

## Abstract

Regulatory T (Treg) cells are essential for maintaining self-tolerance and regulating immune responses. In this study, we report the identification of Treg cell epitopes in human α-tubulin that were capable of enhancing IL-10-producing Foxp3^+^ Treg cells and LAG-3^+^CD49b^+^FoxP3^−^ Tr1 cells in vitro, using human peripheral blood mononuclear cells. Similarly, we also demonstrate that a peptide pool containing the identified Treg cell epitopes (αTBL pool) suppressed the T cell responses elicited by HLA class I- and class II-restricted T cell epitopes. Moreover, stimulation of naive CD4^+^ T cells with autologous monocyte-derived dendritic cells in the presence of the αTBL pool promoted the differentiation of functional FoxP3^+^ Treg cells, which suppressed the proliferation of CD3/CD28-activated T cells. Finally, we show that one of the identified epitopes, identical between human and mouse, also stimulated FoxP3^+^ Treg cells in splenocytes isolated from C57BL/6 mice. Considering the elevated expression of α-tubulin in all cell types, the presence of Treg cell epitopes in this protein may facilitate a broad mechanism of immune regulation. Moreover, α-tubulin Treg cell epitopes may prove useful in creating novel treatments for conditions marked by excessive or misdirected immune responses.

## 1. Introduction

Regulatory T (Treg) cells play a critical role in maintaining self-tolerance and modulating immune responses, thus preventing the development of autoimmune and allergic diseases [1]. There are several types of T cells with regulatory activity. However, the most relevant and numerous Treg cells comprise CD4^+^ T cells that express the master transcriptional factor FoxP3 along with CD25. These Treg cells can be classified as thymic-derived Treg (tTreg) and peripheral Treg (pTreg) cells [2]. While tTreg cells develop in the thymus from CD4^+^ T cell precursors and recognize self-antigens [3], pTreg cells differentiate in the periphery from CD4^+^FoxP3^−^T cells and are thought to recognize foreign antigens [4]. However, it has been reported that both tTreg and pTreg cells can originate from T cells with identical T cell receptor (TCR) specificity, as shown in OVA-TCR transgenic OTII mice [5]. Phenotypically, tTreg and pTreg cells are much alike; however, tTreg cells appear to express higher levels of Helios and Neuropilin 1 (Nrp1) [6], a distinction that remains controversial [7]. Another relevant subset of Treg cells includes type 1 regulatory T (Tr1) cells, which are characterized by the absence of FoxP3 expression and their capacity to produce high levels of interleukin-10 (IL-10). Tr1 cells can be distinguished from other FoxP3^−^ T cells by the co-expression of the surface markers integrin α-2 (also known as ITGA2 or CD49b) and lymphocyte activation gene 3 (LAG-3) [8].

FoxP3^+^ Treg cells require TCR stimulation provided by the recognition of specific Treg cell epitopes to become activated. These epitopes are presented by major histocompatibility class II (MHC II) molecules—human leukocyte antigen class II (HLA II) molecules in humans—on the surface of antigen-presenting cells (APCs) [9]. Upon activation, Treg cells inhibit conventional effector T cells regardless of their antigen specificity, resulting in bystander immunosuppression [10,11,12]. Moreover, Treg cells can also target other immune system cells, including antigen-presenting cells like B cells, macrophages and dendritic cells (DCs). Treg cells exert immunosuppression through contact-dependent and independent mechanisms [10,11]. For instance, Treg cells can inhibit DCs in a contact-dependent manner by interacting with CD80/86 and MHC II molecules through the inhibitory receptors CTLA-4 and LAG-3, respectively. Conversely, Treg cells release inhibitory cytokines including IL-10, IL-35 and TGF-β, which inhibit the activity of T cells and dendritic cells (DCs) in a manner that does not require direct contact. Additionally, Treg cells deprive effector T cells of IL-2, which they take it from the surroundings via their high-affinity IL-2 receptor [10,11].

Despite much research on Treg cells, their precise antigen specificity remains obscure and only a few Treg cell epitopes have been described. De Groot and colleagues pioneered the discovery of Treg cell epitopes, identifying them in the Fc region of immunoglobulin G (IgG) and coining the term Tregitope [13]. These researchers directed their attention to IgG because of the known immunomodulatory properties of intravenous immunoglobulin (IVIG) treatments [14]. Subsequently, Treg cell epitopes have been reported in other self-antigens, including a prostate-specific antigen [15], Factor V protein [16] and low-density lipoprotein receptor-related protein 1 (LRP1) [17]. In contrast, Treg cell epitopes in foreign antigens have been rarely reported. A search in the Immune Epitope Database (IEDB) [18], the largest epitope repository, reveals only a few Treg cell epitopes in foreign sources with suppression activity: three from human cytomegalovirus (two in phosphoprotein 65 antigen and one in immediate early protein IE1), which also activated effector T cells [19], and another in SARS-CoV-2, mapping in replicase polyprotein 1ab [20].

In this study, we based our research on the knowledge that intestinal nematodes release excretory/secretory (ES) products that can promote immunosuppression by inducing Treg cells [21]. Interestingly, ES products encompass numerous antigens that bear resemblance to host proteins [22]. Therefore, we hypothesized that these antigens might create a false sense of self and could include Treg cell epitopes. Following this hypothesis, we examined the existence of Treg cell epitopes in ES antigens derived from common human intestinal nematodes (hINs) that exhibit complete identity with human antigens. As a result, we identified conserved Treg cell epitopes in human α-tubulin that can induce FoxP3^+^ Treg cells and have immunosuppressive capacity. Using different approaches, we demonstrated that α-tubulin Treg cell epitopes suppressed T cell responses induced by epitope peptide antigens. More importantly, we showed that α-tubulin Treg cell epitope peptides can induce the differentiation of naive CD4^+^ T cells into functional FoxP3^+^ Treg cells. Finally, we found that one of the identified α-tubulin Treg cell epitopes with predicted binding to mouse MHC class II molecules also stimulated splenic Treg cells from C57BL/6 mice in vitro. The potential significance of α-tubulin Treg cell epitopes in regulating immune responses will be discussed.

## 2. Results

### 2.1. Prediction and Selection of Potential Human Treg Cell Epitopes

**Figure 1:**
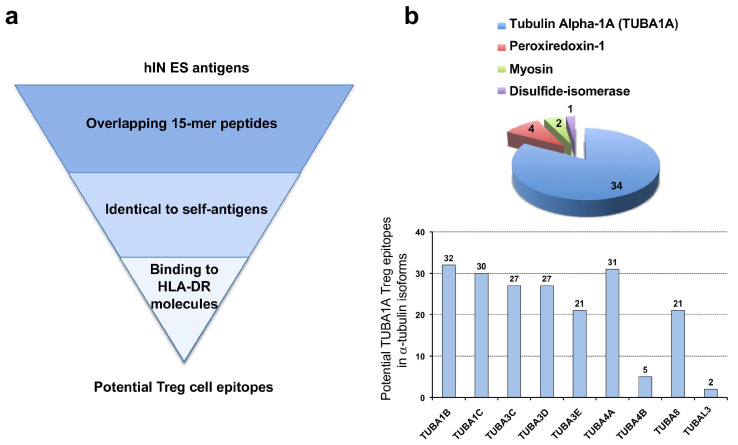
Discovery of potential Treg cell epitopes. (**a**) Strategy for in silico Treg cell epitope discovery. Treg cell epitopes were identified using ES antigens from common human intestinal nematodes (hIN) as bait, selecting peptides identical to human proteins and with predicted binding to HLA-DR molecules. (**b**) Summary of Treg cell epitope discovery results. The pie chart depicts the number of potential Treg cell epitopes identified and their antigen source. Most of the potential Treg cell epitopes were identified in tubulin alpha-1A (TUBA1A) but are also present in other α-tubulin protein isoforms encoded by the genes noted in the graph.

We sought to discover epitopes recognized by CD4^+^FoxP3^+^ regulatory T (Treg) cells, using ES antigens from common human intestinal nematodes (hINs), including *Ascaris lumbricoides*, *Trichuris trichiura*, *Necator americanus* and *Ancylostoma duodenale*, as bait. We focused on ES antigens since they are instrumental to nematodes in the induction of immunosuppression by enhancing the host Treg cells [21]. We specifically considered ES antigen peptides that were identical to human proteins and had predicted binding to HLA-DR molecules as potential Treg cell epitopes (Figure 1a). Briefly, we first assembled a dataset consisting of 47 ES antigens from hINs. Next, overlapping 15-mer peptides (10-residue overlap) covering the entire ES antigens were generated and used as a query in BLASTP searches against human proteins encoded by housekeeping genes. Subsequently, unique peptides with 100% identity to self-antigens were subjected to HLA II binding predictions. As a result of this analysis, we identified 95 peptides in ES-antigens with 100% identity to self-antigens, of which 41 were predicted to bind to at least one of the targeted HLA-DR molecules (Supplementary Table S1). Interestingly, the vast majority of potential Treg cell epitopes identified through this approach were found in tubulin alpha 1A chain (α-tubulin 1A) (Figure 1a). Thus, among the 41 potential Treg cell epitopes—those with predicted binding to HLA-DR molecules—34 were from α-tubulin 1A (TUBA1A), while the remaining were from peroxiredoxin-1 (four peptides), myosin (two peptides) and disulfide-isomerase (one peptide) (Figure 1b).

In humans, there are several isoforms of α-tubulin proteins encoded by different genes that share extensive sequence similarity [23]. As result, the great majority of potential Treg cell epitopes that were anticipated in TUBA1A are also present in α-tubulin proteins encoded by TUBA1B, TUBA1C, TUBA3C, TUBA3D, TUBA3E, TUBA4A and TUBA8, without a single amino acid change (Figure 1 and Supplementary Table S2), and virtually all are conserved with a percentage of identity > 90% (Supplementary Table S2). Accordingly, we will refer to these epitopes as α-tubulin Treg cell epitopes throughout this manuscript.

The population coverage of all the potential α-tubulin Treg cell epitopes reaches 80.93%, as computed by the IEDB coverage tool (http://tools.iedb.org/population, accessed on 10 March 2024). This coverage represents the percentage of the population that will be able to respond to the Treg cell epitopes, considering their HLA-DR binding profiles. However, such coverage can be reached with far fewer peptides, as some of the potential α-tubulin Treg cell epitopes displayed promiscuous binding to several HLA-DR molecules. In Table 1, we show nine potential α-tubulin Treg cell epitope peptides that were predicted to bind to four or more HLA-DR molecules. It is worth noting that some of the predicted epitopes have overlapping sequences and HLA-DR binding profiles. Therefore, we selected seven epitope candidates for experimental scrutiny, based on a rationale aimed at enhancing the likelihood of identifying Treg responses to distinct Treg cell epitopes. This rationale rested on two main criteria: (1) the selection of the most promiscuous peptides and (2) the selection of non-overlapping peptides (<9-residue overlap), unless they exhibit different HLA-DR binding profiles, as they may correspond to the same Treg cell epitope. The binding of peptides to HLA II molecules relies mostly on a nine-mer binding core [24], thus the choice of nine residues to define the overlap. Moreover, if two epitope candidates were overlapping (≥9-residue overlap) and possessed identical HLA-DR binding profiles, the peptide with a greater number of charged residues was chosen for experimental examination. Accordingly, peptide FV_395_ was excluded from experimentation since it overlaps with peptide LV_391_, which possesses an identical HLA-DR binding profile and contains a greater number of charged residues (Table 1). Similarly, peptide AA_389_ was not selected for experimental analysis as it overlaps with peptide LV_391_ and its HLA-DR binding profile does not include any HLA-DR molecule that is absent from the HLA-DR binding profile of peptide LV_391_ (Table 1). Overall, the selected seven potential α-tubulin Treg cell epitopes have the same population coverage as the entire set of potential Treg cell epitopes (80.93%).

### 2.2. In Vitro Validation of α-Tubulin Treg Cell Epitopes in Humans

**Figure 2:**
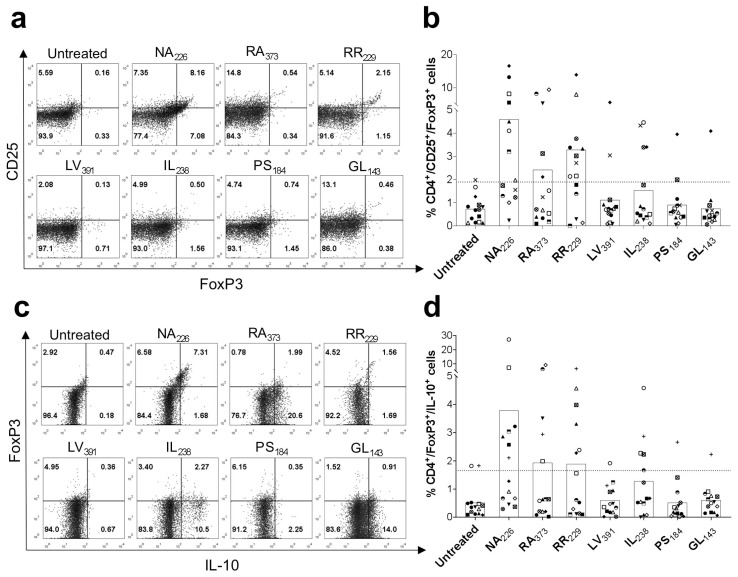
In vitro validation of α-tubulin Treg cell epitopes. PBMCs from 14 subjects were stimulated with selected α-tubulin peptides (NA_226_, RA_373_, RR_229_, LV_391_, IL_238_, PS_184_ and GL_143_) in RPMI complete medium in the presence of IL-2 for 6 days. As controls, cells were cultured without peptides (medium with 0.3% DMSO) (Untreated). Subsequently, cells were stained to analyze CD4^+^CD25^+^FoxP3^+^ and CD4^+^FoxP3^+^IL-10^+^ cells. (**a**) Representative dot plot showing the percentage of CD4^+^CD25^+^FoxP3^+^ cells in response to selected α-tubulin peptides. Cells gated on CD4-positive cells. (**b**) Percentage of CD4^+^CD25^+^FoxP3^+^ cells determined for each donor (**c**) Representative dot plot showing the percentage of CD4^+^FoxP3^+^IL-10^+^ cells in response to selected α-tubulin peptides. Cells gated on CD4-positive cells. (**d**) Percentage of CD4^+^FoxP3^+^IL-10^+^ cells in each donor. Plots in panels (**a**,**c**) correspond to the same donor. In panels (**b**,**d**), the dotted horizontal line marks the threshold that was used for considering positive responses (>meanUntreated + 2 × SDUntreated), each symbol represents a different donor and the bars represent mean values.

To validate the predicted α-tubulin Treg cell epitope peptides, we studied their capacity to stimulate Treg cells found in PBMCs. To that end, PBMCs obtained from 14 healthy donors were stimulated with the individual predicted Treg cell epitope peptides for 6 days. Subsequently, CD4^+^CD25^+^FoxP3^+^ and CD4^+^FoxP3^+^IL-10^+^ cell populations were evaluated by flow cytometry. A positive response was considered when a given peptide induced an increase in the percentage of Treg cells exceeding the mean value of the untreated group plus two standard deviations. Treg cell responses to the predicted Treg cell epitope peptides varied widely among different donors. However, the responses were particularly consistent for peptides NA_226_, RA_373_, RR_229_ and IL_238_ (Figure 2). These four peptides enhanced CD4^+^CD25^+^FoxP3^+^ and CD4^+^FoxP3^+^IL-10^+^ cells above the set threshold in more than three subjects (Figure 2b,d) and were combined in a peptide pool (αTBL pool) for further studies. Furthermore, we incorporated the peptide LV_391_ into the αTBL peptide pool. When analyzing the data in relation to the untreated condition, it was observed that this peptide, as well as NA_226_, RA_373_, RR_229_ and IL_238_, significantly increased Treg cells by more than threefold in a minimum of three donors, as shown in a preliminary preprint version of this study [25]. Moreover, incubation of PBMCs with this peptide also resulted in the stimulation of TGF-β-producing FoxP3^+^ Treg cells (Supplementary Figure S1). Similarly, the remaining peptides included in the αTBL pool, with the exception of IL_238_, also stimulated TGF-β-producing FoxP3^+^ Treg cells in vitro, with a positive response as defined above in at least two of six subjects tested (Supplementary Figure S1).

### 2.3. Tr1 Cell Activation by α-Tubulin Peptides

**Figure 3:**
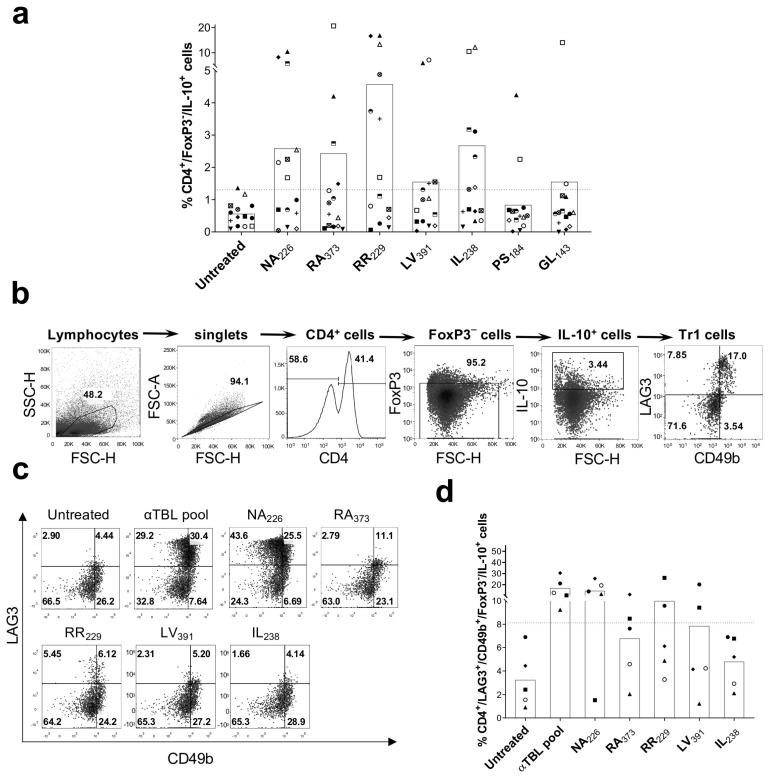
Tr1 cell activation by α-tubulin peptides. (**a**) Percentage of IL-10-producing CD4^+^FoxP3^−^ cells in the PBMCs and conditions shown in Figure 2. (**b**) Gating strategy to identify CD4^+^LAG-3^+^CD49b^+^FoxP3^−^IL-10^+^ cells in PBMCs. (**c**) Representative dot plot showing CD4^+^LAG-3^+^CD49b^+^FoxP3^−^IL-10^+^ cells in PBMCs stimulated with α-tubulin peptides NA_226_, RA_373_, RR_229_, LV_391_ and IL_238_, αTBL pool or without peptides (medium with 0.3% DMSO) (Untreated). (**d**) Percentage of CD4+LAG-3^+^CD49b^+^FoxP3^−^IL-10^+^ cells induced in the noted conditions (*n* = 5). The dotted horizontal line marks the threshold that was set for positive responses (>meanUntreated + 2 × SDUntreated), each symbol represents a different donor and the bars represent mean values.

In the experiments described above, we noted that α-tubulin peptides also increased the numbers of IL-10-producing FoxP3^−^CD4^+^ T cells in some donors, as exemplified in Supplementary Figure S2 for peptide RR_229_. Specifically, peptides NA_226_, RA_373_, RR_229_, IL_238_ and LV_391_ increased the percentage of IL-10^+^FoxP3^−^CD4^+^ cells substantially (>meanUntreated + 2 × SDUntreated) in more than three subjects (Figure 3a). These results suggest that α-tubulin Treg cell epitopes may activate FoxP3^−^Treg cells like Tr1 cells. To explore this possibility, we stimulated PBMCs from five healthy donors with α-tubulin Treg cell epitopes as indicated previously (6-day cultures in the presence of IL-2) and after the relevant staining looked at the CD4^+^LAG^−^3^+^CD49b^+^FoxP3^−^IL-10^+^ Tr1 cell population (Figure 3b). As shown in Figure 3c,d, all the peptides, with the exception of IL_238_, appear to enhance CD4^+^LAG^−^3^+^CD49b^+^FoxP3^−^IL-10^+^ Tr1 cells in some of the subjects, with the peptide NA_226_ inducing the strongest response in most subjects (Figure 3d). Similarly, the αTBL pool, containing peptides NA_226_, RA_373_, RR_229_, IL_238_ and LV_391_, enhanced this Tr1 cell population substantially in all five subjects.

### 2.4. Coverage and Magnitude of α-Tubulin-Specific FoxP3^+^ Treg Cell Responses

**Figure 4:**
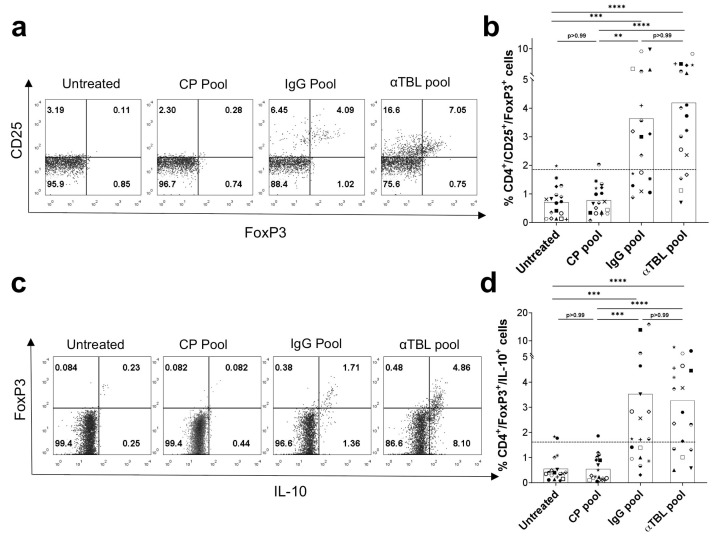
Treg cell responses to a peptide pool with α-tubulin Treg cell epitopes. PBMCs from 18 donors were stimulated with the αTBL pool for 6 days. As controls cells were induced with the IgG pool, CP pool or without peptides (0.3% DMSO) (Untreated). Finally, CD25^+^FoxP3^+^ cells and FoxP3^+^IL-10^+^ cells were evaluated by flow cytometry on CD4-positive gated lymphocytes. (**a**) Representative dot plot showing CD4^+^CD25^+^FoxP3^+^ cells in different conditions. Cells gated on CD4-positive cells. (**b**) Percentage of CD4^+^CD25^+^FoxP3^+^ cells stimulated by different peptide pools in different donors. (**c**) Representative dot plot showing CD4^+^FoxP3^+^IL-10^+^ cells in the different conditions. Gate is on CD4-positive cells. (**d**) Percentage of CD4^+^FoxP3^+^IL-10^+^ cells stimulated by different peptide pools in different donors. In panels (**b**,**d**), the dotted horizontal line marks the threshold that was used for positive responses (>meanUntreated + 2 × SDUntreated), each symbol represents a different donor and the bars represent mean values. Statistically significant differences between conditions were obtained by applying Kruskal–Wallis tests followed by post hoc Dunn’s tests and significant *p*-values are shown as follows: *p* ≤ 0.01 (**), *p* ≤ 0.001 (***) and *p* ≤ 0.0001 (****).

The identified α-tubulin Treg cell epitopes included in the αTBL peptide pool could be expected to induce Treg cell responses in 79.58% of the world population (population coverage), as computed after the gene frequencies of the relevant HLA-DRB molecules that were predicted to bind these epitopes (Table 1). To get an estimation of the actual population coverage of the αTBL pool, we stimulated PBMCs from 18 subjects with the αTBL pool and subsequently evaluated CD4^+^CD25^+^FoxP3^+^ and CD4^+^FoxP3^+^IL-10^+^ T cell populations as previously described. As negative and positive peptide controls, PBMCs were incubated with control self-peptides (CP pool) and known Treg cell epitopes from IgG (IgG pool), respectively. The CP pool consists of self-peptides from human C3 complement protein with predicted promiscuous binding to HLA-DR molecules (sequences in Material and Methods). C3 complement protein is highly expressed in sera and lack of response to the CP pool verifies the specificity of positive responses. As shown in Figure 4, there was a positive response in CD4^+^CD25^+^FoxP3^+^ (Figure 4a,b) and CD4^+^FoxP3^+^IL-10^+^ (Figure 4c,d) cells to the αTBL pool in most donors, 77.77% and 72.22%, respectively. Regardless of the donor, the increase in CD4^+^CD25^+^FoxP3^+^ and CD4^+^FoxP3^+^IL-10^+^ cells upon αTBL stimulation was statistically significant compared to untreated cells and the CP pool (*p* < 0.0001). There was no statistical difference between the αTBL pool and the IgG pool in increasing CD4^+^CD25^+^FoxP3^+^ and CD4^+^FoxP3^+^IL-10^+^ Treg cells.

### 2.5. Suppression of T Cell Responses by α-Tubulin Treg Cell Epitopes

**Figure 5:**
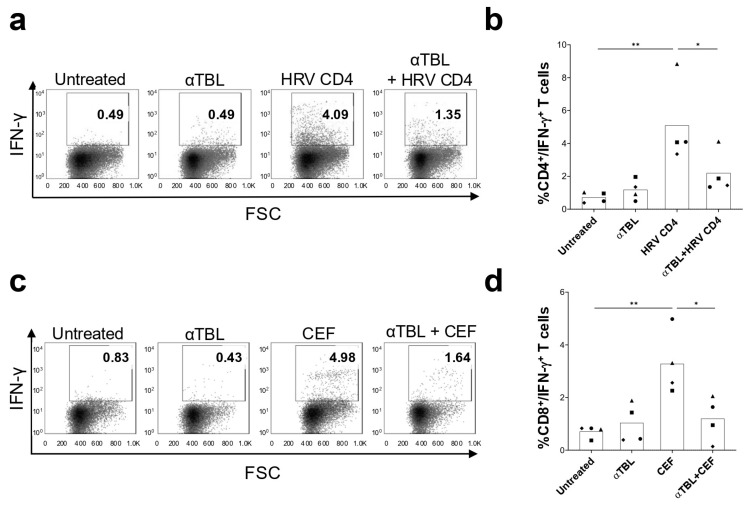
Immunosuppression of antigen-specific T cell responses by α-tubulin Treg cell epitopes. PBMCs from four healthy donors were stimulated with HRV CD4 pool or CEF pool with or without αTBL pool for 6 days in the presence of IL-2. As a control, cells were cultured without peptides (0.3% DMSO) (Untreated). Cells were stained extracellularly with anti-CD3 antibody in combination with anti-CD8 or anti-CD4 antibodies and intercellularly with IFN-γ antibody and analyzed by flow cytometry. (**a**) Representative dot plot showing IFN-γ^+^ cells on CD3 and CD4-positive cells under different conditions. (**b**) Percentage of CD4^+^IFN-γ^+^ T cells in different conditions (*n* = 4). (**c**) Representative dot plot showing IFN-γ^+^ cells on CD3 and CD8 positive cells under different conditions. (**d**) Percentage of CD8^+^IFN-γ^+^ T cells in different conditions (*n* = 4). In panels (**b**,**d**), each symbol represents a different donor, and bars represent mean values. Significant differences were obtained by applying One-way ANOVA tests followed by post hoc Tukey tests and shown as follows: *p* ≤ 0.05 (*) and *p* ≤ 0.01 (**).

We studied the capacity of α-tubulin Treg cell epitopes (αTBL pool) to suppress CD4^+^ and CD8^+^ T cell responses induced by the HRV CD4 and CEF peptide pools, respectively. To that end, PBMCs were incubated with the HRV CD4 or the CEF peptide pools alone or in combination with the αTBL pool for 6 days and subsequently analyzed IFN-γ-producing CD4^+^ T cells (HRV CD4 pool stimulus) or CD8^+^ T cells (CEF pool stimulus) by flow cytometry (details in Material and Methods). As shown in Figure 5, stimulation with HRV peptides increased IFN-γ-producing CD4^+^ T cells, which were significantly reduced in the presence of the αTBL pool (*p* < 0.05) (Figure 5a,b). Likewise, stimulation of PBMCs with the CEF pool (CD8^+^ T cell epitopes) increased IFN-γ-producing CD8^+^ T cells, and in the presence of the αTBL pool, this cell population was significantly reduced (*p* < 0.05) (Figure 5c,d).

### 2.6. α-Tubulin Treg Cell Epitopes Induce Functional FoxP3^+^ Treg Cells from Naive T Cells

**Figure 6:**
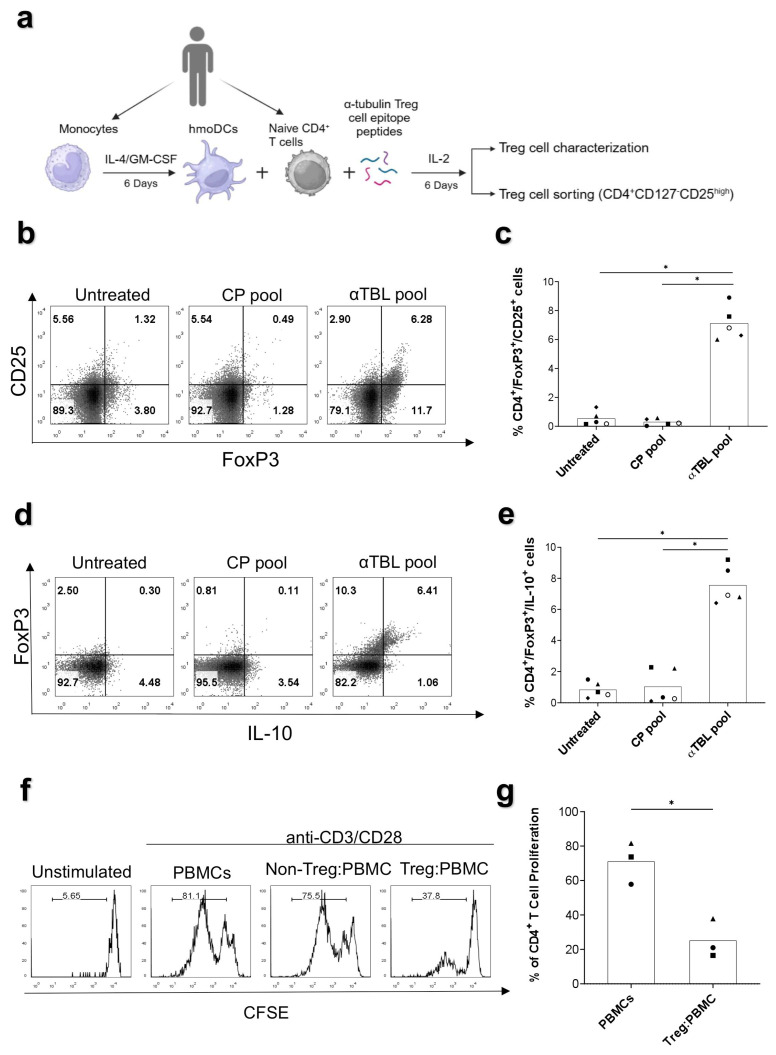
Differentiation and characterization of functional α-tubulin-specific Treg cells. (**a**) Experimental procedure to differentiate α-tubulin-specific FoxP3^+^ Treg cells. moDCs were differentiated from monocytes isolated from PBMCs by culturing them with IL-4 and GM-CSF for 6 days. moDCs were then co-cultured with autologous naive CD4^+^ T cells during 6 days in the presence of IL-2 and the αTBL peptide pool. As a control, co-cultures were incubated with the CP pool and without peptides (0.3% DMSO) (Untreated). Peptide pools and IL-2 were renewed every 2 days. Subsequently, Treg cells in the different co-cultures were evaluated by flow cytometry (panels (**b**–**d**)) and Treg cells were sorted and used in bystander suppression assays panels (**f**,**g**). (**b**) Representative dot plot showing CD4^+^CD25^+^FoxP3^+^ cells differentiated in co-cultures of moDCs and naive CD4^+^ T cells. (**c**) Plot depicting the percentage of CD4^+^CD25^+^FoxP3^+^ cells differentiated under different conditions (*n* = 5). (**d**) Representative dot plot showing CD4^+^FoxP3^+^IL-10^+^ cells differentiated in co-cultures of moDCs and naive CD4^+^ T cells. (**e**) Plot depicting the percentage of CD4^+^FoxP3^+^IL-10^+^ cells differentiated under different conditions (*n* = 5). (**f**) Bystander inhibition of cell proliferation by α-tublin-specific Treg cells. Purified FoxP3^+^ Treg cells were cultured with allogeneic CFSE-labeled PBMCs and stimulated with anti-CD3/CD28 Dynabeads. CFSE-dilution assay was used to measure T cell proliferation. Histograms show CFSE-staining in CD4-positive cells in non-stimulated PBMCs (Unstimulated) and CD3/CD28-stimulated PBMCS alone (PBMCs) or in culture with Treg cells (Treg:PBMC) and non-Treg cells (non-Treg:PBMCs). (**g**) Plot showing the percentage of T cells that proliferated after CD3/CD28 stimulation in different donors (*n* = 3). Each symbol represents a different donor and the bars represent mean values. Significant differences were obtained by applying student *t*-tests and shown as follows: *p* ≤ 0.05 (*).

We also investigated whether α-tubulin Treg cell epitopes can induce the differentiation of FoxP3+ Treg cells from naive CD4^+^ T cells. To that end, naive CD4^+^ T cells isolated from PBMCs (Supplementary Figure S3) were co-cultured for 6 days with autologous moDCs in the presence of an IL-2 and αTBL pool (Figure 6a). As controls, moDC-T cell co-cultures were incubated without peptides (Untreated) or with a CP pool (control self-peptides from C3 complement protein). Subsequently, CD4^+^CD25^+^FoxP3^+^ and CD4^+^FoxP3^+^IL-10^+^ cells were analyzed by flow cytometry. As shown in Figure 6b–e, the percentage of CD4^+^CD25^+^FoxP3^+^ and CD4^+^FoxP3^+^IL-10^+^ cells showed a remarkable increase in response to the αTBL pool, reaching approximately 8% of all CD4^+^ T cells. In contrast, the CP pool did not increase these cell populations. We also observed that a significant fraction of CD4^+^FoxP3^+^ T cells induced by the αTBL pool expressed Npr1 and Helios, as shown in Supplementary Figure S4.

To determine whether these Treg cells were functional, we isolated them by cell sorting (cell sorting strategy provided in Supplementary Figure S5) and subsequently evaluated their ability to suppress the proliferation of T cells using a CFSE-dilution assay. As shown in Figure 6f,g, CD4^+^CD127^low/−^CD25^high^ (Treg) but not CD4^+^CD127^+^CD25^−^ (non-Treg) cells obtained from moDC-T cell co-cultures with the αTBL pool, suppressed the proliferation of CD3/CD28-stimulated CD4^+^ T cells in PBMCs. Overall, the results show the capacity of α-tubulin Treg cell epitopes to differentiate functional Treg cells from peripheral naive CD4^+^ T cells.

### 2.7. Selection and Validation of α-Tubulin Treg Cell Epitopes in Mice

**Figure 7:**
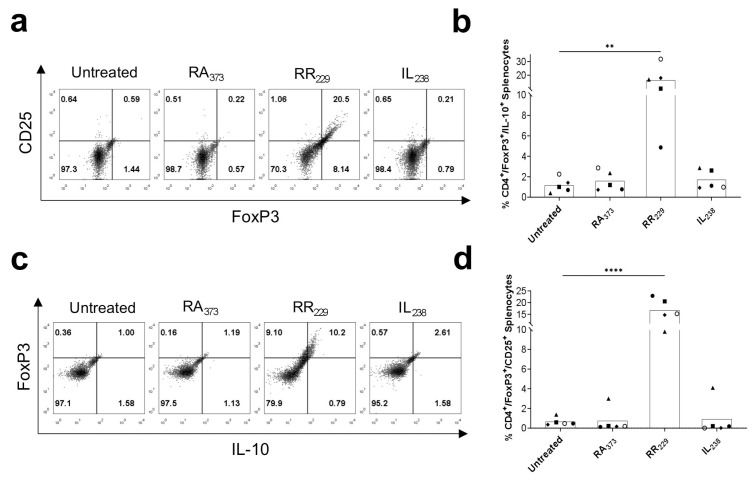
Treg cell epitope validation in C57BL/6 mice. Splenocytes from five C57BL/6 mice were stimulated with α-tubulin Treg cell epitope peptides, predicted to bind to I-Ab (RA_373_, RR_229_ and IL_238_ peptides). Splenocytes were cultured during 3 days in RPMI complete medium in the presence of IL-2. As a control, cells were cultured without peptides (medium with 0.3% DMSO) (Untreated). Subsequently, cells were stained to analyze CD4^+^CD25^+^FoxP3^+^ and CD4^+^FoxP3^+^IL-10^+^ cells by flow cytometry. (**a**) Representative dot plot showing the percentage of CD25^+^FoxP3^+^ cells in different conditions. Gate is on CD4-positive cells. (**b**) Percentage of CD4^+^CD25^+^FoxP3^+^ splenocytes in mice in different conditions (*n* = 5). (**c**) Representative dot plot showing the percentage of FoxP3^+^IL-10^+^ cells in different conditions. Gate is on CD4-positive cells. (**d**) Percentage of CD4^+^FoxP3^+^IL-10^+^ splenocytes in mice in different conditions (*n* = 5). Significant differences were determined using One-way ANOVA tests and shown as follows: *p* ≤ 0.01 (**) and *p* ≤ 0.0001 (****).

We verified that the α-tubulin peptides NA_226_, RA_373_, RR_229_ and IL_238_ that more clearly stimulated Treg cells in humans are entirely conserved in mice: 100% sequence identity to mouse α-tubulin proteins as corroborated by BLAST searches (details in Material and Methods). Moreover, peptides RA_373_, RR_229_ and IL_238_ were predicted to bind to I-Ab, which is the MHC II molecule expressed by C57BL/6 mice. Therefore, we evaluated these peptides as potential Treg cell epitopes in C57BL/6 mice. To that end, splenocytes obtained from five C57BL/6 mice were stimulated with the selected peptides for 3 days in the presence of IL-2. As a control, splenocytes were cultured with medium alone (Untreated). Subsequently, cells were stained and CD4^+^CD25^+^FoxP3^+^ and CD4^+^FoxP3^+^IL-10^+^ Treg cell populations were analyzed by flow cytometry. As shown in Figure 7, stimulation with peptide RR_229_ enhanced both CD4^+^CD25^+^FoxP3^+^ (Figure 7a,b) and CD4^+^FoxP3^+^IL-10^+^ cells (Figure 7c,d). Specifically, CD4^+^CD25^+^FoxP3^+^ and CD4^+^FoxP3^+^IL-10^+^ cells increased from about 1% in untreated cells to more than 16% in the condition with peptide RR_229_. Based on these findings, peptide RR_229_ can be considered an α-tubulin Treg cell epitope in C57BL/6 mice and could be used for future in vivo studies.

## 3. Discussion

Treg cells become activated upon the recognition of specific Treg cell epitopes. However, identifying Treg cell epitopes is far from trivial and there are few well-characterized Treg cell epitopes. The majority of Treg cell epitopes have been found in self-antigens [13,15,16,17] and are likely recognized by tTreg cells, which represent the major subset of Treg cells in blood [3]. In this work, we sought to discover Treg cell epitopes through a computer-assisted strategy depicted in Figure 1a. The strategy consisted in identifying potential Treg cell epitopes as peptides shared between ES antigens from human intestinal nematodes (hINs) and self-antigens with predicted binding to HLA-DR molecules. This approach primarily identified peptides belonging to human α-tubulin (Figure 1b). Subsequently, we investigated seven potential Treg cell epitopes with predicted promiscuous binding to HLA-DR molecules and verified that at least four of them (NA_226_, RA_373_, RR_229_ and IL_238_) (Table 1) stimulated CD4^+^CD25^+^FoxP3^+^ and IL-10-producing FoxP3^+^ Treg cells in PBMCs obtained from different subjects (Figure 2a–d). We also found that these α-tubulin peptides, with the exception of IL_238_, can stimulate TGF-β-producing FoxP3^+^ Treg cells (Supplementary Figure S1). In these experiments, we assume that α-tubulin peptides can directly stimulate Treg cells upon HLA II presentation by the APCs found in the PBMCs. However, further confirmatory studies involving the inhibition of HLA II presentation may be warranted. Conversely, since we only tested a limited set of α-tubulin peptides, we cannot rule out the presence of additional Treg cell epitopes in α-tubulin proteins. Likewise, we cannot discard that peptides identified in peroxiredoxin-1 (four peptides), myosin (two peptides) and disulfide-isomerase (one peptide), which also met our selection criteria, may represent Treg cell epitopes.

Interestingly, we found evidence that α-tubulin peptides may also be recognized by FoxP3^−^ Treg cells (Figure 3). In particular, stimulation of PBMCs with peptides NA_226_ and RR_229_ resulted in a substantial enhancement of IL-10-producing CD4^+^LAG-3^+^CD49b^+^FoxP3^−^ Tr1 cells (Figure 3c,d). Tr1 cells represent a major group of IL10-producing FoxP3^−^ Treg cells that have a major contribution in peripheral tolerance by limiting excessive inflammation [26]. However, the activation of Tr1 cells by α-tubulin peptides may also be a bystander consequence of the stimulation of FoxP3^+^ Treg cells, particularly through their cytokine production. Indeed, Tr1 cells are very responsive to the environmental milieu of cytokines and especially to the presence of IL-10 [26]. Tr1 cells express the IL-10 receptor (IL-10R), and IL-10R signaling is essential to sustain the regulatory activity of Tr1 cells in vivo and in vitro [27]. Moreover, IL-10 in combination with IFN-α has also been shown to promote the differentiation of Tr1 cells in vitro [28]. Therefore, additional research including tetramer assays is necessary to confirm the direct recognition of α-tubulin peptides by both FoxP3^+^ Treg cells and Tr1 cells.

Judging by their predicted HLA-DR binding profiles, the identified α-tubulin Treg cell epitopes could be recognized by most people (79.58% population coverage). Indeed, we verified that a peptide pool comprising these α-tubulin Treg cell epitopes (αTBL pool) enhanced CD4^+^CD25^+^FoxP3^+^ Treg cells in 14 out of 18 donors (77.7%) and CD4^+^FoxP3^+^IL-10^+^ Treg cells in 13 donors (72.2%), comparable to the responses detected to the IgG pool, comprising two verified Treg cell epitopes identified in IgG (Figure 4a–d). Considering that the peptides in the αTBL pool exhibit overlapping HLA-DR binding profiles, the same population coverage may be reached with a smaller number of peptides.

We also found that α-tubulin peptides exhibit immunosuppressive capacity, which is likely linked to the stimulation of FoxP3^+^ Treg cells and Tr1 cells. Thus, the αTBL pool suppressed T cell responses induced by HLA I- and HLA II-restricted peptide antigens (Figure 5a–d). While competition for HLA II molecules was not ruled out as a mechanism that could account for the suppression of CD4^+^ T cell responses, it is important to emphasize that α-tubulin peptides also suppressed CD8^+^ T cells responses stimulated by HLA I-restricted epitopes (CEF pool). On the other hand, competition for HLA binding is unlikely to serve as an effective means of immunosuppression, as there are mechanisms that facilitate the presentation of low-affinity and low-abundant peptides [29,30,31]. Furthermore, experimental data suggest that even a single peptide–MHC complex per cell is sufficient to trigger a T cell response [32]. Collectively, these findings indicate the existence of Treg cell epitopes within α-tubulin that can stimulate functional Treg cells, which in turn inhibit T cells irrespective of their antigen specificity. However, it will be necessary to conduct mechanistic experiments to strengthen this claim. Indeed, as these results were obtained using PBMCs, other mechanisms of immunosuppression that do not involve Treg cells cannot be ruled out.

Considering the experimental design, the FoxP3^+^ Treg cells that responded to α-tubulin peptides are likely tTreg cells. However, additional experiments will be required to define the characteristics of α-tubulin specific FoxP3^+^ Treg cells in PBMCs. tTreg cells can actually co-exist with pTreg cells, which have many similarities; however, pTreg cells develop in the periphery from naive CD4^+^ T cells and are thought to recognize foreign antigens [4]. Interestingly, we found that the co-culture of naive CD4^+^ T cells with moDCs in the presence of α-tubulin peptides (αTBL pool) induced functional FoxP3^+^ Treg cells that were capable of suppressing the proliferation of CD3/CD28-stimulated T cells (Figure 6a–f). As shown in Supplementary Figure S3, naive CD4^+^ T cells co-cultured with moDCs did not express FoxP3. Hence, it is unlikely that the Treg cells produced in these experiments derived from contaminating FoxP3^+^ Treg cells. Instead, we propose that Treg cells differentiated in vitro from α-tubulin auto-reactive naive CD4^+^ T cells that have escaped negative selection and may have a propensity to become Treg cells. Indeed, research has shown that the preferential source of pTreg cells in mice consists of recent thymic emigrants that possess an inherent tendency to acquire a FoxP3^+^CD25^+^ phenotype [33]. Similarly, pTreg cells recognizing α-tubulin are likely to develop in vivo as well. Indeed, the observation that pTreg and tTreg cells share similar TCR repertoires [34] supports that pTreg cells may recognize the same self-antigens as tTreg cells, consequently reducing the necessity for Treg cell epitopes in foreign antigens. Interestingly, a significant fraction of FoxP^+^ Treg cells (~18%) generated in the co-culture experiments displayed elevated levels of Npr1 and Helios (Supplementary Figure S4) and may resemble tTreg cells, despite being induced in vitro. Early research in mice suggested that high Npr1 and Helios expression correlated with tTreg cells [6]. However, in humans, Helios and Nrp1 expression levels do not reliably distinguish tTreg cells from pTreg cells, and their levels may merely reflect activation status rather than origin [35]. Stimulation of conventional CD4 T cells in the presence of TGF-β1 and IL-2 can also induce the generation of FoxP3^+^ Treg cells in vitro [7]. Therefore, it will be worth exploring whether TGF-β1 has also a role in the induction of FoxP3^+^ Treg cells from naive CD4 T cells in our co-culture experiments.

Treg cells are important not only to avoid immune reactions against self-antigens but also to control excessive immune responses and inflammation. Indeed, the stimulation of conventional effector T cells is concomitant with that of Treg cells [7]. In this scenario, the presence of Treg cell epitopes in α-tubulin has significant implications. α-tubulin forms heterodimers with β-tubulin, which polymerize into microtubules, forming the main component of the cytoskeleton [36]. Consequently, all cells express significant amounts of α-tubulin, and during an immune response APCs shall display MHC II molecules with bound peptides derived from both α-tubulin and foreign antigens. Although α-tubulin is a cytoplasmic protein, it is well documented that APCs can present endogenous antigens by MHC II molecules [37]. Indeed, research has demonstrated that a considerable fraction of the MHC II peptidome originates from cytosolic proteins, including in the context of inflammation [38,39]. As a result, α-tubulin-specific Treg cells recruited to the inflammatory site could be activated by APCs and regulate the immune response.

Given the high conservation of α-tubulin across mammals [40] and the evolutionary stability of adaptive immune mechanisms [41], α-tubulin Treg cell epitopes likely contribute to immune regulation and homeostasis across species. Indeed, we confirmed that one of the α-tubulin Treg cell epitopes identified in humans (peptide RR_229_) enhanced CD4^+^CD25^+^FoxP3^+^ and IL-10-producing FoxP3^+^ Treg cells in C57BL/6 mice in vitro utilizing splenocytes (Figure 7). Subsequently, the prophylactic and therapeutic potential of this α-tubulin Treg cell epitope could be studied in vivo using C57BL/6 mice. Treg cell epitopes are indeed promising immunotherapeutic agents, as already shown for IgG-derived Treg cell epitopes in preclinical animal models of inflammatory and autoimmune diseases, including asthma [42,43], inflammatory bowel disease (IBD) [44] and type 1 diabetes (T1D) [45]. We would expect that α-tubulin Treg cell epitopes may be also effective in these disease models. Currently, we aim to assess the therapeutic effectiveness of peptide RR_229_ in a C57BL/6 mouse model of acute colitis induced by dextran sulphate sodium [46], administering the peptide either intraperitoneally or sublingually. Similarly, we intend to evaluate the therapeutic potential of this Treg cell epitope in a C57BL/6 mouse model of allergic airway inflammation induced by the house dust mite (HDM) [47]. It is essential to emphasize that the functionality, stability and safety of the induced Treg cells must also be examined in vivo before proceeding with any potential clinical application of the identified α-tubulin Treg cell epitopes.

## 4. Materials and Methods

### 4.1. The Identification of Excretory–Secretory Antigens from Prevalent Human Enteric Nematodes

A dataset of protein antigens in ES products from prevalent human intestinal nematodes (hINs), including *Ascaris lumbricoides*, *Trichuris trichiura*, *Necator americanus and Ancylostoma* duodenale, was assembled as follows. ES proteins from nematodes, regardless of species, were first identified through text mining protein and literature records at NCBI, and their amino acid sequences were downloaded in FASTA format. Next, CD-HIT [48] was used to discard redundant amino acid sequences (identity threshold of 90%). The resulting non-redundant proteins, assembled into a single FASTA file, were subsequently used as a query for remote BLAST searches [49] at NCBI, limiting the results to hIN organisms (command line: blastp -remote -query non_redundant_nematode_es_proteins.fasta -db nr -query -entrez_query “Ascaris lumbricoides | Trichuris trichiura | Necator americanus | Ancylostoma duodenale [Organism]” -evalue 1e-20 -num_alignments 10). Protein hits with ≥80% identity were selected as hIN ES proteins and amino acid sequences collected in a FASTA file. Redundant amino acid sequences were then discarded using CD-HIT [48] (identity threshold of 90%). As a result, a dataset consisting of the amino acid sequence of 47 hIN ES proteins in FASTA format was obtained. The FASTA file can be provided by the corresponding author upon written request.

### 4.2. Prediction of Treg Cell Epitopes and Population Coverage

Treg cell epitopes were anticipated in hIN ES proteins based on (a) identity to human proteins (self-antigens) and (b) binding to human leukocyte antigens class II (HLA II molecules). To identify peptides in hIN ES proteins shared by human self-antigens, overlapping 15-mer peptides with a 10-residue overlap covering the entire amino acid sequences of hIN ES proteins were generated. Subsequently, these peptides were used as queries in sequence similarity searches against human proteins encoded by housekeeping genes using BLASTP v2.13.0 [49]. Housekeeping genes were those reported by Eisenberg and Levanon [50]. BLAST searches were performed with default parameters, but the e-value was set to 10,000. BLAST results were processed, and peptide hits from non-gapped 15-residue length alignments of 100% identity to self-antigens were selected and targeted for binding predictions to HLA II molecules.

The binding of selected 15-mer peptides to selected HLA class II (HLA II) molecules and to mouse MHC class II (MHC II) molecule I-Ab was predicted using a standalone version of NetMHCII v2.2 [51], setting the input to peptides. HLA II-peptide binding analysis were limited to HLA-DR molecules encompassing the following beta chains: HLA-DRB1*01:01, HLA-DRB1*03:01, HLA-DRB1*04:01, HLA-DRB1*04:04, HLA-DRB1*04:05, HLA-DRB1*07:01, HLA-DRB1*08:02, HLA-DRB1*09:01, HLA-DRB1*11:01, HLA-DRB1*13:02, HLA-DRB1*15:01, HLA-DRB3*01:01, HLA-DRB4*01:01 and HLA-DRB5*01:01. HLA-DR molecules incorporate a non-polymorphic α chain and the selected β chains are expressed by ~80% of the population as computed by IEDB coverage tool (http://tools.iedb.org/population/, accessed on 10 March 2024) [52]. For human HLAII molecules, only strong binders were considered for further functional assays, while for mouse MHC II molecules, weak binders were also considered.

### 4.3. Peptides and Peptide Pools

Predicted Treg cell epitope peptides, IgG Treg cell epitope peptides, Human Rhinovirus (HRV)-specific CD4^+^ T cell epitope peptides and control self-peptides from complement C3 protein were purchased from ProteoGenix (Schiltigheim, France) at a 2 mg scale with a purity level ≥ 90%. IgG peptides consisted of the core sequence of two experimentally verified human Treg cell epitopes derived from the Fc region of IgG (LQSSGLYSLSSVVTVPSSSL and YNSTYRVVSVLTVLH) [13]. HRV peptides consisted of seven conserved HLA II-restricted CD4^+^ T cell epitope peptides from HRV (VKDVLEKGIPTLQSPTVE, DSTITSQDVANAVVGYGV, VANAVVGYGVWPHYLTPE, INLRTNNSSTIVVPYIN, KEKFRDIRRFIP and GLEPLDLNTSAGFPYV, DLPYVTYLKDELR) [53]. Control self-peptides consisted of five 15-mer peptides (LRLPYSVVRNEQVEI, KAAVYHHFISDGVRK, ISKYELDKAFSDRNT, VNFLLRMDRAHEAKI, PEGIRMNKTVAVRTL) from human complement C3 protein (GenBank accession: AAI50180.1) predicted to bind to at least four different HLA-DR molecules. Peptide binding predictions to HLA-DR molecules were carried out as described above. Synthetic peptides were provided lyophilized and were reconstituted in 80% dimethyl sulfoxide (DMSO) and diluted to a final stock concentration of 8 mM (40% DMSO). The following custom synthetic peptide pools were prepared (1 mM of each peptide): the αTBL pool, consisting of α-tubulin Treg cell epitope peptides identified in this research; the IgG pool, containing the two IgG Treg cell epitope peptides; the CP pool, including the control self-peptides from human complement C3 protein; and the HRV CD4 pool, comprising HRV peptides. All custom synthetic peptides alone or combined in pools were used at a final concentration of 10 µM (each peptide) in cell cultures and the concentration of DMSO did not exceed 0.3%. The CEF pool, comprising 23 HLA I-restricted immunodominant CD8^+^ T cell epitope peptides from human cytomegalovirus, Epstein–Barr virus and influenza virus, was purchased from Mabtech (Nacka Strand, Sweden) and reconstituted in DMSO plus phosphate-buffered saline (PBS) buffer (200 µg/mL final concentration), following the manufacturer’s instructions.

### 4.4. Isolation of Peripheral Blood Mononuclear Cells, Monocytes and Naive CD4^+^ T Cells

Peripheral blood mononuclear cells (PBMCs) were isolated from buffy coats by a density gradient on Ficoll-Paque Plus (Sigma-Aldrich, Darmstadt, Germany). PBMCs within the interface layer were carefully collected, subjected to 2 washes with cold PBS by centrifugation at 300× *g* for 5 min, and resuspended in RPMI 1640 medium (Gibco, Waltham, MA, USA) supplemented with 10% heat-inactivated human serum (Gibco, Waltham, MA, USA), 2 mM L-glutamine (Lonza, Visp, Switzerland), 100 U/mL penicillin (Lonza, Visp, Switzerland) and 100 μg/mL streptomycin (Lonza, Visp, Switzerland) (RPMI complete medium). Buffy coats were provided by the regional blood transfusion center (Centro de Transfusion de la Comunidad de Madrid, Spain) and were obtained from consenting healthy blood donors. Donors signed an informed consent document, following current legislation as described in the Royal Decree-Law 1088/2005 of September 16 (BOE-A-2005-15514). Monocytes were isolated from PBMCs through magnetic separation with CD14 MicroBeads (Miltenyi Biotec, Bergisch Gladbach, Germany), following the manufacturer’s instructions. About 5 × 10^6^ of CD14^+^ cells were obtained from 50 × 10^6^ PBMCs. Naive CD4^+^ T cells were also isolated from PBMCs utilizing the MojoSort™ Human Naive CD4 T Cell Isolation Kit (BioLegend, San Diego, CA, USA) in accordance with the manufacturer’s guidelines. On average, 5 × 10^6^ naive CD4^+^ T cells were isolated from 5 × 10^7^ PBMCs. The purity and phenotype of freshly isolated naive CD4^+^ T cells was analyzed by flow cytometry after staining the cells with anti-human CD4 (MEM-241, APC, Immunotools, Friesoythe, Germany), anti-human CD45RA (HI100, PE, BD Biosciences, Franklin Lakes, NJ, USA) and anti-human CD45RO (UCHL1, FITC, Miltenyi Biotech, Bergisch Gladbach, Germany) antibodies.

### 4.5. Isolation of Splenocytes from C57BL/6 Mice

Male C57BL/6 mice (8–12 weeks old) were obtained from Charles River and were housed at the animal facility of the Faculty of Medicine, Complutense University of Madrid. Experiments were approved by the Ethics Committee of the Complutense University of Madrid and were conducted in accordance with the applicable legislation on animal experimentation (D.C. 86/609/CEE; RD 1201/2005). Mice were sacrificed by cervical dislocation, under general anesthesia with 1–2% isoflurane/O_2_. At termination, spleens were aseptically removed, minced and filtered through 70 μm nylon cell strainers to obtain a single-cell suspension. Cells were washed with cold PBS containing 2% of inactivated fetal bovine serum (FBS) (Gibco, Waltham, MA, USA) and cellular splenic suspensions were prepared after hypotonic lysis of erythrocytes in ammonium-chloride-potassium (ACK) lysis buffer (Gibco, Waltham, MA, USA). Splenocytes were then washed twice with cold PBS containing 2% FBS. Erythrocyte-free splenocytes were resuspended in RPMI complete medium, including 10% of FBS instead of human serum and 50 μM of β-mercaptoethanol (Sigma-Aldrich, Darmstadt, Germany). Cells were quantified using Trypan blue in a Neubauer chamber under a light microscope.

### 4.6. In Vitro Validation of Treg Cell Epitopes in Humans

Treg cell epitopes were validated using fresh PPMCs from 14 healthy blood donors. PBMCs were cultured at 2 × 10^6^ cells per well in 24-well plates (Corning, Corning, NY, USA) using 1000 µL of RPMI complete medium supplemented with IL-2 10 U/mL (Immunotools, Friesoythe, Germany) and stimulated with individual peptides or peptide pools (10 µM per peptide). As a control, cells were incubated without peptides, in medium with 0.3% DMSO (Untreated cells). Plates were incubated at 37 °C in 5% CO_2_ for 6 days. Peptides and IL-2 were renewed every 2 days, and 200 μL of growth medium was replenished. After 6-day cultures, cells were stimulated with 50 ng/mL phorbol 12-myristate 13-acetate (PMA) (Sigma Aldrich, Darmstadt, Germany) and 1 μg/mL ionomycin (Sigma Aldrich, Darmstadt, Germany) in the presence of 10 μg/mL Brefeldin A (Thermo Fisher, Waltham, MA, USA) for 4 h at 37 °C in 5% CO_2_. Next, cells were washed with PBS and surface stained with anti-CD4 (MEM-241, FITC, Immunotools, Friesoythe, Germany) antibody alone or with anti-CD25 (MEM-181, APC, Immunotools, Friesoythe, Germany) antibody. Subsequently, cells were fixed, permeabilized and stained intracellularly with anti-FoxP3 (236A/E7, PE, BD Biosciences (Franklin Lakes, NJ, USA) or 3G3, APC, Immunotools (Friesoythe, Germany)) antibody in combination with anti-IL-10 (JES3-19F1, APC, BD Biosciences, Franklin Lakes, NJ, USA) or anti-TGF-β1 (TW4-gE7, PE, BD Biosciences, Franklin Lakes, NJ, USA) antibodies and then analyzed by flow cytometry (FACSCalibur, BD Biosciences, Franklin Lakes, NJ, USA) to detect CD4^+^CD25^+^FoxP3^+^, CD4^+^FoxP3^+^IL-10^+^ and CD4^+^FoxP3^+^TGF-β^+^ Treg cells. Similarly, Tr1 cells were detected in PBMCs stimulated as previously indicated after extracellular stainings with antibodies anti-CD4 (SK3, APC/Cyanine7, Biolegend, San Diego, CA, USA), anti-CD49b (P1E6-C5, PE/Cyanine7, Biolegend, San Diego, CA, USA) and anti-LAG-3 (11C3C65, BV650, Biolegend, San Diego, CA, USA), followed by intracellular stainings with antibodies anti-FoxP3 (236A/E7, PE, BD Biosciences, Franklin Lakes, NJ, USA) and anti-IL-10 (JES3-19F1, APC, BD Biosciences). Detection was performed using flow cytometry (FACSCelesta, BD Biosciences, Franklin Lakes, NJ, USA).

### 4.7. In Vitro Validation of Treg Cell Epitopes in Mice

Selected Treg cell epitopes were validated using fresh splenocytes isolated from five mice. Splenocytes from C57BL/6 were plated in 24-well plates (2 × 10^6^ cells/well) and incubated in RPMI complete medium, including 10% of heat inactivated FBS and 50 μM of β-mercaptoethanol and 10 ng/mL of recombinant mouse IL-2 (Immunotools, Friesoythe, Germany). Cells were stimulated with individual peptides (10 µM/peptide) and plates were incubated at 37 °C with 5% CO_2_ for 3 days. As a control, cells were incubated without peptides, in medium with 0.3% DMSO (Untreated cells). After the 3-day incubation, cells were stimulated with 50 ng/mL PMA and 1 μg/mL ionomycin in the presence of 10 μg/mL Brefeldin A (Thermo Fisher, Waltham, MA, USA) for 4 h at 37 °C in 5% CO_2_. Then, cells were washed with PBS and surface stained using anti-CD4 (GK1.5, FITC, Immunotools, Friesoythe, Germany) and anti-CD25 (PC61.5.3, APC, Immunotools, Friesoythe, Germany) antibodies. Next, cells were fixed, permeabilized and stained intracellularly with antibodies anti-FoxP3 (3G3, PE, Immunotools, Friesoythe, Germany) and anti-IL-10 (JES5-16E3, APC, BD Biosciences, Franklin Lakes, NJ, USA). Finally, cells were acquired on a FACSCalibur flow cytometer (BD Biosciences, Franklin Lakes, NJ, USA) and CD4^+^CD25^+^FoxP3^+^ and CD4^+^FoxP3^+^IL-10^+^ cell populations analyzed.

### 4.8. Treg Cell Epitope Immunosuppression Assays

The capacity of validated Treg cell epitope peptides (αTBL pool) to suppress T cell responses to antigen-specific stimuli (CEF pool and HRV CD4 pool) was measured as follows. Freshly isolated PBMCs were plated at 1 × 10^6^ cells per well in 48-well plates on RPMI complete medium (500 µL) and stimulated with the HRV CD4 peptide pool (10 μM each peptide) or with the CEF pool (2 µg/mL) either alone or in the presence of the αTBL pool (10 μM each peptide) in the presence of 10 U/mL of IL-2. As a control, cells were incubated without peptides, in the presence of 0.3% DMSO (Untreated cells). Plates were incubated at 37 °C in 5% CO_2_ for 6 days. Peptides and IL-2 were renewed every 2 days, replenishing 100 μL of growth medium. Next, intracellular IFN-γ staining assays were carried out to quantify IFN-γ-producing T cells. Briefly, cells were incubated for 16 h at 37 °C in 5% CO_2_ with 5 μg/mL of Brefeldin A (Thermo Fisher, Waltham, MA, USA) and then surface stained with antibodies anti-CD3 (UCHT-1, APC, Immunotools, Friesoythe, Germany) and anti-CD4 (MEM-241, FITC, Immunotools, Friesoythe, Germany) (conditions with HRV CD4 pool) or anti-CD8 (HIT8a, FITC, Immunotools, Friesoythe, Germany) (conditions with CEF pool). Subsequently, cells were fixed, permeabilized and stained intracellularly with anti-IFN-γ antibody (B27, PE, Immunotools, Friesoythe, Germany). Finally, cells were acquired and analyzed by flow cytometry (FACSCalibur flow cytometer, BD Biosciences, Franklin Lakes, NJ, USA).

### 4.9. Generation of Monocyte-Derived Dendritic Cells and Co-Culture with Naive T Cells

Monocyte-derived dendritic cells (moDCs) were generated by culturing monocytes in 48-well plates (1 × 10^6^ cells/well) with 500 µL of RPMI complete medium per well, supplemented with IL-4 (Immunotools, Friesoythe, Germany) and granulocyte–macrophage colony-stimulating factor (GM-CSF) (Immunotools, Friesoythe, Germany) at a concentration of 100 ng/mL each. Cells were incubated at 37 °C and 5% CO_2_ for 6 days. Cytokines were renewed at day 4, and 100 μL of growth medium was replenished every 2 days. moDCs obtained as described were plated in 48-well plates at a cellular density of 0.2 × 10^6^ cells/well, together with purified autologous naive CD4^+^ T cells at a ratio of 1:5 (moDC: naive CD4^+^ T cells) in 500 μL of RPMI complete medium. The α TBL pool or CP pool (control self-peptides) (10 µM/peptide) and IL-2 (10 U/mL) were added to co-cultures on days 0 and 4 of the experiment, and 100 μL of growth medium was replenished every 2 days. As a control, cells were incubated without peptides, in the presence of 0.3% DMSO (Untreated cells). Plates were incubated at 37 °C with CO_2_ for 6 days. Subsequently, cells were harvested, washed with PBS and subjected to surface and intracellular staining with antibodies anti-CD4 (SK3, APC/Cyanine7, Biolegend, San Diego, CA, USA), anti-CD25 (MEM-181, APC, Immunotools, Friesoythe, Germany), anti-FoxP3 (206D, BV421, Biolegend), anti-IL-10 (JES3-9D7, PE, Biolegend, San Diego, CA, USA), anti-Helios (22F6, FITC, Biolegend, San Diego, CA, USA) and anti-Nrp1 (12C2, BV650, Biolegend, San Diego, CA, USA). Finally, cells were acquired on a FACSCelesta flow cytometer (BD Biosciences, Franklin Lakes, NJ, USA).

### 4.10. Bystander Treg Cell Immunosuppression Assay

Naive CD4^+^ T cells co-cultured with moDCs in the presence of the αTBL pool as indicated above were collected and washed with PBS by centrifugation at 300× *g* for 5 min. Cells were counted and about 15 × 10^6^ cells were stained with antibodies anti-CD4 (SK3, APC/Cyanine7, Biolegend, San Diego, CA, USA), anti-CD25 (M-A251, APC, BD Biosciences, Franklin Lakes, NJ, USA) and anti-CD127 (A019D5, PE, Biolegend, San Diego, CA, USA). Next, Treg cells (CD4^+^CD127^low/−^CD25^high^) were sorted by fluorescence-activated cell sorting (FACS) using a FACSAria III cell separator cytometer (BD Biosciences, Franklin Lakes, NJ, USA). The sorting was conducted under aseptic conditions to maintain sterility throughout the process. It was performed in purity mode, which prioritizes obtaining a highly purified population, typically resulting in purities of greater than 95%. This mode minimizes contamination from unwanted cell populations, ensuring a highly pure population of CD4^+^CD127^low/−^CD25^high^ Treg cells. About 0.5 × 10^6^ CD4^+^CD127^low/−^CD25^high^ cells were obtained from 15 × 10^6^ cells, yielding about 3.3% of the starting population. On the other hand, PBMCs from a second subject were obtained and stained with Carboxyfluorescein Diacetate Succinimidyl Ester (CFSE) (Biolegend, San Diego, CA, USA) by incubating 10^7^ PBMCs with 0.5 µM CSFE for 20 min in PBS at 37 °C. CFSE-labeled cells were washed twice using RPMI complete medium by centrifugation at 300× *g* for 5 min and plated in 96-well plates (0.2 × 10^6^ cells/well) together with the purified CD4^+^CD127^low/−^CD25^high^ cells in a 1:1 ratio using 200 μL of RPMI complete medium. Subsequently, cells were stimulated with human T cell activator CD3/CD28 Dynabeads (Gibco, Waltham, MA, USA) following the manufacturer’s instructions and incubated at 37 °C and 5% CO_2_ for 6 days. As controls, CFSE-labeled PBMCs were cultured alone, with or without CD3/CD28 stimulation, and CD4^+^CD127^+^CD25^−^ cells (Non-Treg cells) collected during cell sorting were also mixed with CFSE-labeled CD3/CD28-stimulated PBMCs. Finally, cells were stained with anti-human CD4 antibody (SK3, APC/Cyanine7, Biolegend, San Diego, CA, USA) and analyzed by flow cytometry (FACSCalibur, BD Biosciences, Franklin Lakes, NJ, USA).

### 4.11. General Flow Cytometry Procedures

Cells were washed prior to any staining with PBS by centrifugation at 300× *g* for 5 min. For surface staining, Fc receptors were blocked with 10 μg/mL of human IgG (Merck, Darmstadt, Germany) for human samples and with mouse IgG (10 µg/mL, Merck, Darmstadt, Germany) for mice samples, incubating for 15 min at 4 °C. Next, cells were washed with PBS and incubated for 30 min in the dark at 4 °C with the relevant antibodies in FACS buffer (PBS supplemented with 1% FBS and 1 mM EDTA) (50 μL final volume/sample). Following a washing step, cells were fixed and permeabilized using the Fixation/Permeabilization solution in the FoxP3 staining buffer set (eBioscience, San Diego, CA, USA), and after a washing step, cells were intracellularly stained in Permeabilization Buffer (eBioscience, San Diego, CA, USA) with the relevant antibodies. After staining, cell samples were washed twice in PBS and resuspended in PBS with 1 mM EDTA (300 μL final volume/sample). Cell data were acquired on FACSCalibur or FACSCelesta flow cytometers (BD Biosciences, Franklin Lakes, NJ, USA) and analyzed using FlowJo v10 software (Tree Star, Ashland, OR, USA). For cell data analysis of PBMCs, lymphocytes were selected on forward (FSC) and side scatter (SSC) and subsequently gated on the relevant surface. The positive gate in intracellular cytokine and FoxP3 staining assays was set using fluorescence minus one (FMO) controls.

### 4.12. Sequence Similarity Analyses and Statistical Procedures

The similarity/conservation of selected peptide sequences in mice were analyzed online at the NCBI BLAST site (https://blast.ncbi.nlm.nih.gov/Blast.cgi, accessed on 15 January 2023) using BLASTP and SWISSPROT as the target database, restricting the search to mice (taxid: 10090).

Statistical analyses were performed using GraphPad Prism 8 (GraphPad Software Inc., San Diego, CA, USA). The normal distribution of data was tested using Shapiro–Wilk tests. Kruskal–Wallis tests followed by post hoc Dunn’s tests were used to identify statistical differences between three or more groups when the data were not normally distributed. When the data followed a normal distribution, one-way analysis of variance (ANOVA) tests were employed, followed by Tukey’s Honest Significant Difference (HSD) test for post hoc comparisons. Additionally, Student *t*-tests were applied to compare means from two groups of data. Differences were considered significant when *p* ≤ 0.05 (*), very significant when *p* ≤ 0.01 (**), highly significant when *p* ≤ 0.001 (***) and extremely significant when *p* ≤ 0.0001 (****).

## 5. Conclusions and Limitations

We have identified α-tubulin Treg cell epitopes with suppressive activity, capable of activating various subsets of Treg cells and inducing the differentiation of FoxP3^+^ Treg cells from naive CD4^+^ T cells. Given the ubiquitous and abundant expression of α-tubulin in all cells, the presentation of α-tubulin-derived peptides to Treg cells may contribute to immune homeostasis and the regulation of immune responses. However, this mechanism will need to be confirmed in vivo. Indeed, it is important to highlight that our study was conducted entirely in vitro and followed a particular experimental methodology. This limitation affects the generalizability of our results and conclusions, necessitating further mechanistic and functional investigations, both in vitro and in vivo. Similarly, further studies will be required to evaluate the functionality, stability and safety of the induced Treg cells in vivo before advancing any potential clinical application of the α-tubulin Treg cell epitopes identified in this study.

## Figures and Tables

**Table 1:** Predicted α-tubulin Treg cell epitopes with promiscuous HLA-DR binding.

Name	Sequence	Start	End	Tested	HLA-DR
LV_391_	LDHKFDLMYAKRAFV	391	405	Y	DRB1:01:01,DRB1:07:01,DRB1:09:01,DRB1:11:01,DRB1:15:01,DRB5:01:01
FV_395_	FDLMYAKRAFVHWYV	395	409	N	DRB1:01:01,DRB1:07:01,DRB1:09:01,DRB1:11:01,DRB1:15:01,DRB5:01:01
RR_229_	RLIGQIVSSITASLR	229	243	Y	DRB1:01:01,DRB1:04:01,DRB1:07:01,DRB1:09:01,DRB1:13:02,DRB5:01:01
IL_238_	ITASLRFDGALNVDL	238	252	Y	DRB1:01:01,DRB1:03:01,DRB1:07:01,DRB1:13:02,DRB3:01:01
RA_373_	RAVCMLSNTTAIAEA	373	387	Y	DRB1:01:01,DRB1:04:01,DRB1:04:04,DRB1:07:01,DRB1:13:02
PS_184_	PYNSILTTHTTLEHS	184	198	Y	DRB1:01:01,DRB1:04:01,DRB1:04:04,DRB1:04:05,DRB1:07:01
NA_226_	NLNRLISQIVSSITA	226	240	Y	DRB1:01:01,DRB1:07:01,DRB1:09:01,DRB1:15:01,DRB4:01:01
GL_143_	GGTGSGFTSLLMERL	143	157	Y	DRB1:01:01,DRB1:04:04,DRB1:04:05,DRB1:09:01
AA_389_	ARLDHKFDLMYAKRA	389	403	N	DRB1:01:01,DRB1:09:01,DRB1:11:01,DRB5:01:01

Peptides were named with regard to α-tubulin chain 1A variant (Accession NP_006000), using the first and last amino acid residue followed by the start position in subscript. Peptides that were synthesized and tested are marked as ‘Y’, while those that were not are marked as ‘N’.

## Data Availability

Data are contained within the article and Supplementary Materials.

## Supplementary Materials

The following supporting information can be downloaded at: https://www.mdpi.com/article/10.3390/ijms26178356/s1.

## Abbreviations

The following abbreviations are used in this manuscript:

ACK	Ammonium–Chloride–Potassium
ANOVA	One-way Analysis of Variance
APC	Antigen-Presenting Cell
CEF	Cytomegalovirus, Epstein–Barr Virus, Influenza Virus (peptide pool)
CFSE	Carboxyfluorescein Succinimidyl Ester
DCs	Dendritic Cells
DMSO	Dimethyl Sulfoxide
ES	Excretory/Secretory
FACS	Fluorescence-Activated Cell Sorting
FBS	Fetal Bovine Serum
Fc	Fragment crystallizable (region of IgG)
FITC	Fluorescein Isothiocyanate
FoxP3	Forkhead Box P3
GM-CSF	Granulocyte–Macrophage Colony-Stimulating Factor
HLA	Human Leukocyte Antigen
HRV	Human Rhinovirus
HSD	Honest Significant Difference
hIN	Human Intestinal Nematode
IEDB	Immune Epitope Database
IFN-γ	Interferon-Gamma
IgG	Immunoglobulin G
IL	Interleukin
IVIG	Intravenous Immunoglobulin
LAG-3	Lymphocyte-Activation Gene 3
LRP1	Low-Density Lipoprotein Receptor-Related Protein 1
moDC	Monocyte-Derived Dendritic Cell
MHC	Major Histocompatibility Complex
Nrp1	Neuropilin 1
OVA	Ovalbumin
PBMC	Peripheral Blood Mononuclear Cell
PBS	Phosphate-Buffered Saline
PMA	Phorbol 12-Myristate 13-Acetate
pTreg	Peripheral Regulatory T Cell
TCR	T Cell Receptor
TGF-β	Transforming Growth Factor Beta
Treg	Regulatory T Cell
Tr1	Type 1 Regulatory T Cell
tTreg	Thymic-Derived Regulatory T Cell
TUBA1A	Tubulin Alpha 1A
