# Supplementary material for: Peptides Derived from α-Tubulin Induce Functional T Regulatory Cells
Source: Int J Mol Sci. 2025 Aug 28;26(17):8356. doi: 10.3390/ijms26178356 (PMC12542834; doi:10.3390/ijms26178356)
Supplement: Supplementary file 1 [file ijms-26-08356-s001.zip › Supplementary_Figure S1.pdf]

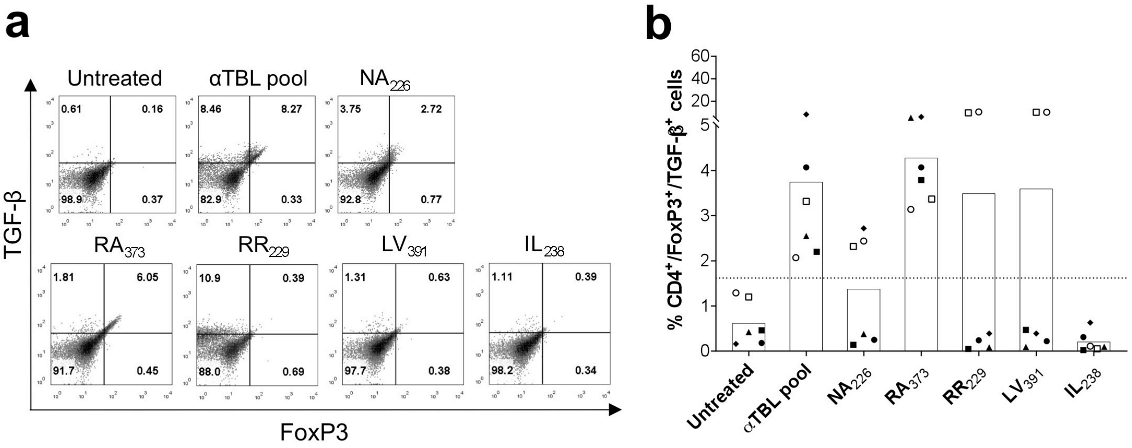

Supplementary Figure S1. CD4^{+}FoxP3^{+}TGF-β^{+} Treg cells induced by selected α-tubulin peptides. (a) Representative dot plot showing the percentage of CD4^{+}FoxP3^{+}TGF-β^{+} cells in response to selected α-tubulin peptides and a peptide pool including all of them (αTBL pool) **(b)** Percentage of CD4^{+}FoxP3^{+}TGF-β^{+} cells for each donor. The dotted horizontal line marks the threshold that was used for positive responses (> $\text{mean}_{\text{Untreated}} + 2 \times \text{SD}_{\text{Untreated}}$), each symbol represents a different donor and bars represent mean values (n=6).
