# Supplementary material for: Peptides Derived from α-Tubulin Induce Functional T Regulatory Cells
Source: Int J Mol Sci. 2025 Aug 28;26(17):8356. doi: 10.3390/ijms26178356 (PMC12542834; doi:10.3390/ijms26178356)
Supplement: Supplementary file 1 [file ijms-26-08356-s001.zip › Supplementary_Figure S2.pdf]

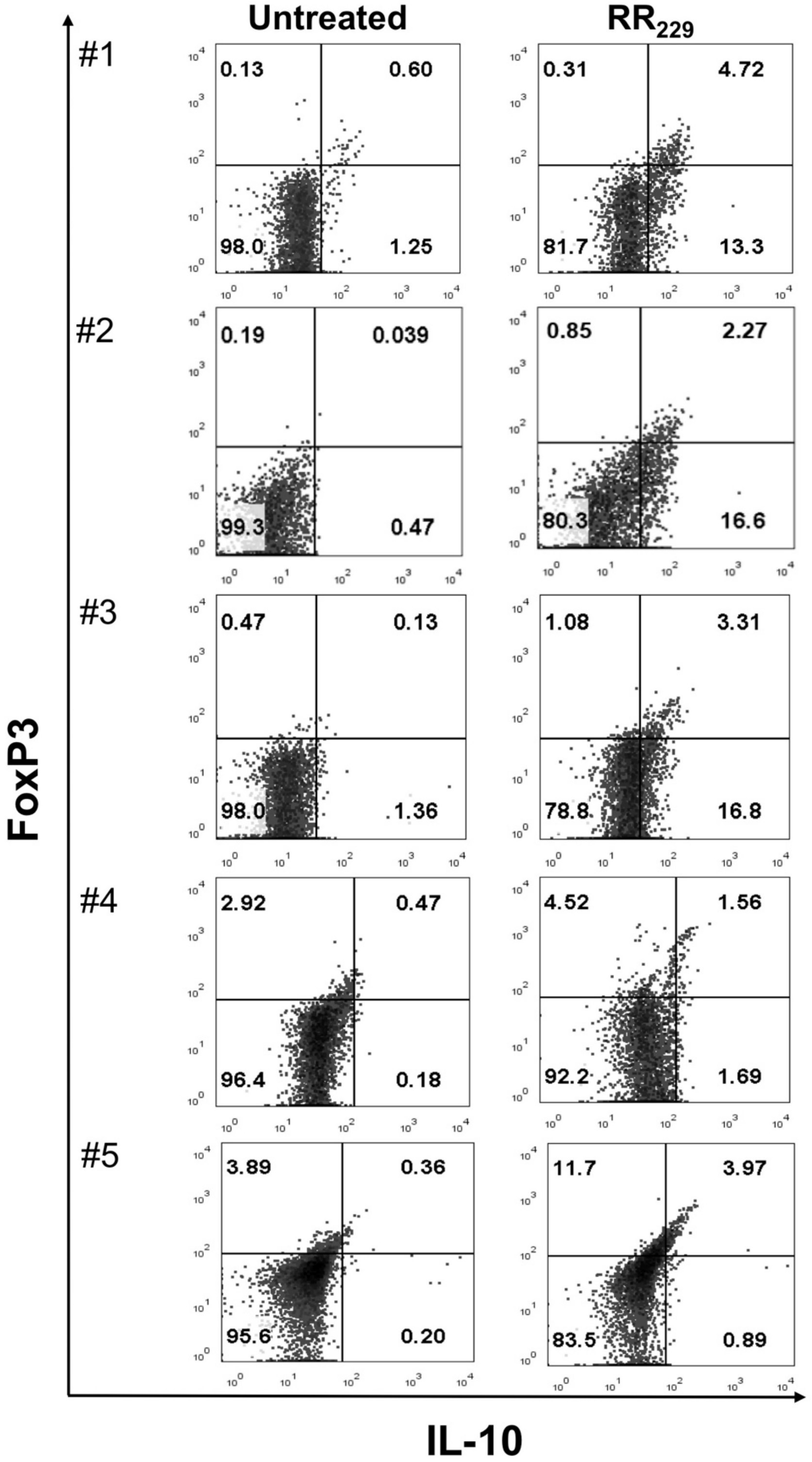

Supplementary Figure S2. IL-10-producing CD4⁺FoxP3⁺ and CD4⁺FoxP3⁻ cells in PBMCs stimulated with RR_{229} peptide. Cytometry dot plots showing human PBMCs from five healthy donors stimulated with the peptide RR_{229} in the presence of IL-2. Cells were stained extracellularly for CD4 and intracellularly for FOXP3 and IL-10, and analyzed by flow cytometry. Cells are gated on CD4 cells. As controls cells were cultured without peptide (Untreated). Donors #1–#4 showed an increase in the frequency of $CD4^{+}FOXP3^{-}$ $IL\text{-}10^{+}$ cells upon RR_{229} stimulation, while donor #5 did not exhibit a notable response.
