# Supplementary material for: Peptides Derived from α-Tubulin Induce Functional T Regulatory Cells
Source: Int J Mol Sci. 2025 Aug 28;26(17):8356. doi: 10.3390/ijms26178356 (PMC12542834; doi:10.3390/ijms26178356)
Supplement: Supplementary file 1 [file ijms-26-08356-s001.zip › Supplementary_Figure S3.pdf]

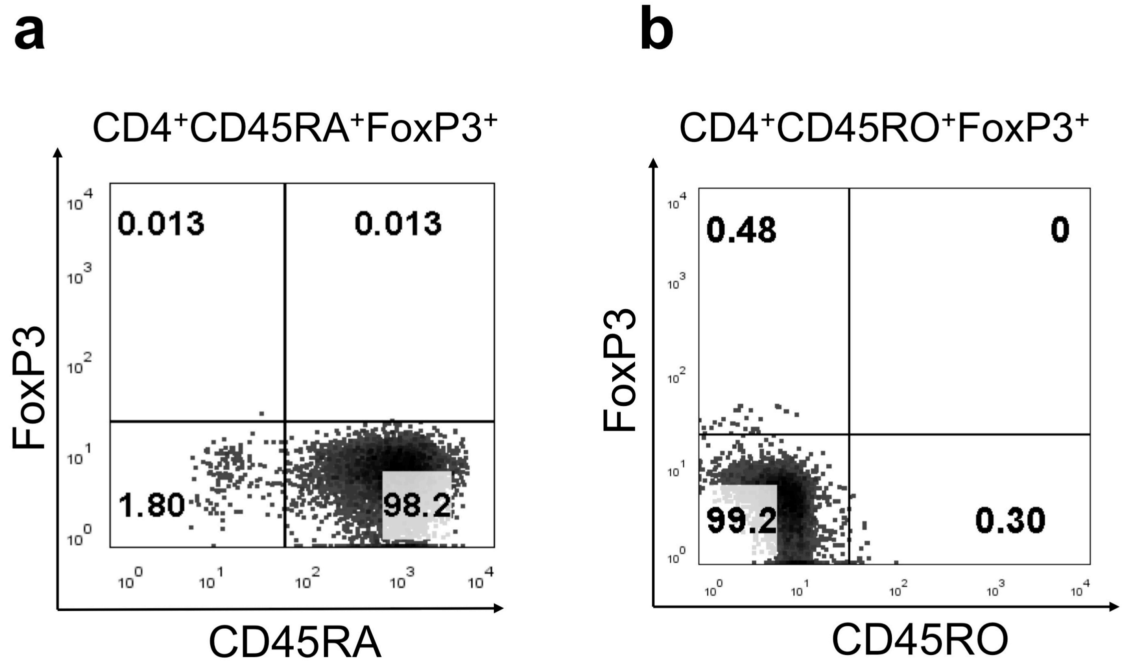

Supplementary Figure S3. Quality control of naive CD4$^+$ T Cells. Dot plots showing CD4$^+$ gated CD45RA$^+$FoxP3$^+$ cells (panel **a**) and CD45RO$^+$FoxP3$^+$ cells (panel **b**) that are present in naive CD4$^+$ T cells isolated from PBMCs using the MojoSort™ Human CD4 Naive T Cell Isolation Kit (BioLegend).
