# Supplementary material for: Peptides Derived from α-Tubulin Induce Functional T Regulatory Cells
Source: Int J Mol Sci. 2025 Aug 28;26(17):8356. doi: 10.3390/ijms26178356 (PMC12542834; doi:10.3390/ijms26178356)
Supplement: Supplementary file 1 [file ijms-26-08356-s001.zip › Supplementary_Figure S4.pdf]

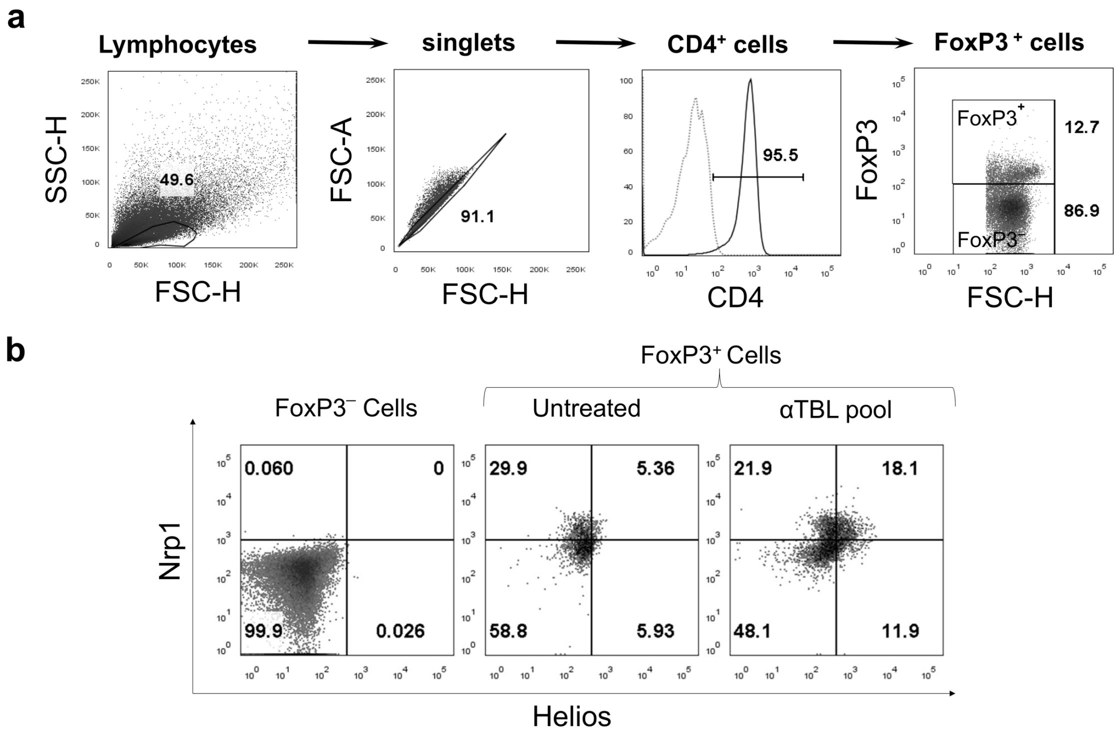

Supplementary Figure S4. CD4$^+$FoxP3$^+$Helios$^+$Nrp1$^+$ Treg cell induced by the αTBL pool. (a) Plot showing the gating strategy used to detect CD4$^+$FoxP3$^+$ Helios$^+$Nrp1$^+$ Treg cells induced after culturing naive CD4$^+$ T cells with moDCs in the presence of the αTBL pool. Lymphocytes were gated based on forward and side scatter, followed by the selection of CD4$^+$ cells. Next, FoxP3$^+$ and FoxP3$^-$ cells were selected, and finally, Helios$^+$Nrp1$^+$ cells were identified within these cell populations. **(b)** Representative dot plot showing CD4$^+$FoxP3$^+$Helios$^+$Nrp1$^+$ Treg cells differentiated in co-cultures of moDCs and naive CD4$^+$ T cells with the αTBL pool and without peptides (Untreated). CD4$^+$FoxP3$^-$ cells of the untreated condition (FoxP3$^-$ cells) were used to gate Helios$^+$Nrp1$^+$ Treg cells.
