# Supplementary material for: Peptides Derived from α-Tubulin Induce Functional T Regulatory Cells
Source: Int J Mol Sci. 2025 Aug 28;26(17):8356. doi: 10.3390/ijms26178356 (PMC12542834; doi:10.3390/ijms26178356)
Supplement: Supplementary file 1 [file ijms-26-08356-s001.zip › Supplementary_Figure S5.pdf]

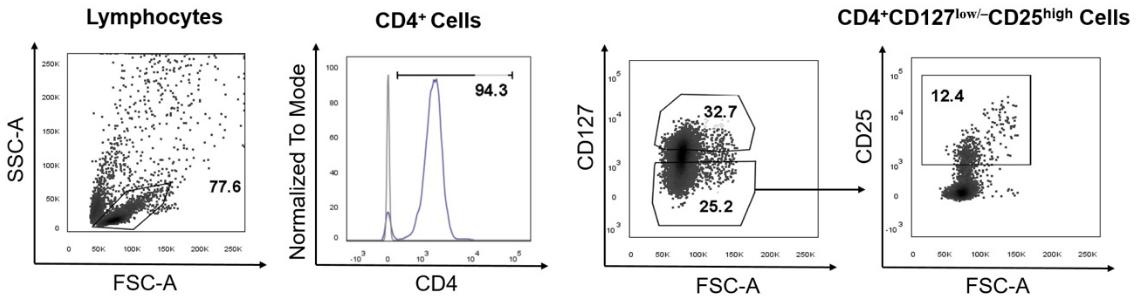

Supplementary Figure S5. Gating strategy used to sort CD4^{+}CD127$^{low/–}$CD25^{high} Treg cells. Plot showing the gating strategy used to isolate CD4^{+}CD127$^{low/–}$CD25^{high} Treg cells differentiated *in vitro* by co-culturing naive CD4^{+} T cells with monocyte derived dendritic cells (moDCs) in the presence of the αTBL pool (details in Material and Methods). Lymphocytes were gated based on forward and side scatter, followed by the selection of CD4^{+} cells. Next, CD127$^{low/–}$ cells were selected, and finally, CD25^{high} cells were identified within the CD4^{+}CD127 $^{low/–}$ population.
