# Supplementary material for: Peptides Derived from α-Tubulin Induce Functional T Regulatory Cells
Source: Int J Mol Sci. 2025 Aug 28;26(17):8356. doi: 10.3390/ijms26178356 (PMC12542834; doi:10.3390/ijms26178356)
Supplement: Supplementary file 1 [file ijms-26-08356-s001.zip › Supplementary_Table S1.pdf]

Supplementary Table S1. Potential Treg cell epitopes: peptides in ES proteins from hINs with 100% identity to human self-antigens and predicted binding to selected HLA-DR molecules

Peptide sequence	ES antigen	hIN Specie	Human Antigen	Antigen name	Predicted HLA II (HLA-DRB) binding profile					
LDHKFDLMYAKRAFV	CDW57876/403-417	Trichuris trichiura	NP_006000	Tubulin alpha-1A	DRB1:01:01	DRB1:07:01	DRB1:09:01	DRB1:11:01	DRB1:15:01	DRB5:01:01
FDLMYAKRAFVHWYV	XP_013298506/391-405	Necator americanus	NP_006000	Tubulin alpha-1A	DRB1:01:01	DRB1:07:01	DRB1:09:01	DRB1:11:01	DRB1:15:01	DRB5:01:01
RLIGQIVSSITASLR	CDW57876/241-255	Trichuris trichiura	NP_006000	Tubulin alpha-1A	DRB1:01:01	DRB1:04:01	DRB1:07:01	DRB1:09:01	DRB1:13:02	DRB5:01:01
ITASLRFDGALNVDL	KIH68283/319-333	Ancylostoma duodenale	NP_006000	Tubulin alpha-1A	DRB1:01:01	DRB1:03:01	DRB1:07:01	DRB1:13:02	DRB3:01:01	
RAVCMLSNTTAIAEA	CDW57876/385-399	Trichuris trichiura	NP_006000	Tubulin alpha-1A	DRB1:01:01	DRB1:04:01	DRB1:04:04	DRB1:07:01	DRB1:13:02	
PYNSILTTHTTLEHS	KIH68283/265-279	Ancylostoma duodenale	NP_006000	Tubulin alpha-1A	DRB1:01:01	DRB1:04:01	DRB1:04:04	DRB1:04:05	DRB1:07:01	
NLNRLIGQIVSSITA	CDW57876/238-252	Trichuris trichiura	NP_006000	Tubulin alpha-1A	DRB1:01:01	DRB1:07:01	DRB1:09:01	DRB1:15:01	DRB4:01:01	
GGTGSGFTSLLMERL	XP_013298506/139-153	Necator americanus	NP_006000	Tubulin alpha-1A	DRB1:01:01	DRB1:04:04	DRB1:04:05	DRB1:09:01		
ARLDHKFDLMYAKRA	XP_013298506/385-399	Necator americanus	NP_006000	Tubulin alpha-1A	DRB1:01:01	DRB1:09:01	DRB1:11:01	DRB5:01:01		
VVEPYNSILTTHTTL	CDW57876/193-207	Trichuris trichiura	NP_006000	Tubulin alpha-1A	DRB1:01:01	DRB1:07:01	DRB5:01:01			
TGSGFTSLLMERLSV	CDW57876/157-171	Trichuris trichiura	NP_006000	Tubulin alpha-1A	DRB1:01:01	DRB1:09:01	DRB1:11:01			
GFTSLLMERLSVDYG	KIH68283/229-243	Ancylostoma duodenale	NP_006000	Tubulin alpha-1A	DRB1:01:01	DRB1:11:01	DRB4:01:01			
TAVVEPYNSILTTHT	XP_013298506/175-189	Necator americanus	NP_006000	Tubulin alpha-1A	DRB1:01:01	DRB1:04:05	DRB1:07:01			
SLRFDGALNVDLTEF	CDW57876/253-267	Trichuris trichiura	NP_006000	Tubulin alpha-1A	DRB1:01:01	DRB1:04:05	DRB1:13:02			
PLDFTFVCPTEIIAF	CDW58032/61-75	Trichuris trichiura	NP_002565	peroxiredoxin-1	DRB1:01:01	DRB1:07:01	DRB1:09:01			
YNSILTTHTTLEHSD	XP_013298506/181-195	Necator americanus	NP_006000	Tubulin alpha-1A	DRB1:01:01	DRB1:04:05	DRB1:07:01			
TGFKVGINYQPPTVV	CDW57876/361-375	Trichuris trichiura	NP_006000	Tubulin alpha-1A	DRB1:01:01	DRB1:07:01	DRB5:01:01			
DCAFMVDNEAIYDIC	CDW57876/211-225	Trichuris trichiura	NP_006000	Tubulin alpha-1A	DRB1:03:01	DRB3:01:01				
LEFSIYPAPQVSTAV	XP_013298506/163-177	Necator americanus	NP_006000	Tubulin alpha-1A	DRB1:01:01	DRB1:09:01				
KLEFSIYPAPQVSTA	KIH68283/247-261	Ancylostoma duodenale	NP_006000	Tubulin alpha-1A	DRB1:01:01	DRB1:09:01				
RTGTYRQLFHPEQLI	CDW57876/91-105	Trichuris trichiura	NP_006000	Tubulin alpha-1A	DRB1:01:01	DRB1:11:01				
FTSLLMERLSVDYGK	XP_013298506/145-159	Necator americanus	NP_006000	Tubulin alpha-1A	DRB1:11:01	DRB4:01:01				
FTFVCPTEIIAFSDR	XP_013307087/505-519	Necator americanus	NP_002565	Peroxiredoxin-1	DRB1:01:01	DRB1:07:01				
YRGDVVPKDVNAAIA	CDW57876/331-345	Trichuris trichiura	NP_006000	Tubulin alpha-1A	DRB1:13:02	DRB3:01:01				
RNWQWWRLFTKVKPL	XP_013291219/823-837	Necator americanus	NP_002464	Myosin	DRB1:07:01	DRB1:11:01				
ILRQITVNDLPVGRS	XP_013307087/595-609	Necator americanus	NP_002565	Peroxiredoxin-1	DRB1:03:01	DRB1:04:05				
EHSDCAFMVDNEAIY	KIH68283/277-291	Ancylostoma duodenale	NP_006000	Tubulin alpha-1A	DRB3:01:01					
SNTTAIAEAWARLDH	CDW57876/391-405	Trichuris trichiura	NP_006000	Tubulin alpha-1A	DRB5:01:01					
VGINYQPPTVVPGGD	XP_013298506/349-363	Necator americanus	NP_006000	Tubulin alpha-1A	DRB1:01:01					
FFYPLDFTFVCPTEI	XP_013307087/499-513	Necator americanus	NP_002565	Peroxiredoxin-1	DRB1:07:01					
FDGALNVDLTEFQTN	KIH68283/325-339	Ancylostoma duodenale	NP_006000	Tubulin alpha-1A	DRB1:03:01					
TNLVPYPRIHFPLAT	XP_013298506/253-267	Necator americanus	NP_006000	Tubulin alpha-1A	DRB1:15:01					
KRAFVHWYVGEGMEE	XP_013298506/397-411	Necator americanus	NP_006000	Tubulin alpha-1A	DRB1:15:01					
TYAPVISAEKAYHEQ	CDW57876/283-297	Trichuris trichiura	NP_006000	Tubulin alpha-1A	DRB5:01:01					
QLRCNGVLEGIRICR	XP_013291219/691-705	Necator americanus	NP_002464	Myosin	DRB5:01:01					
QTNLVPYPRIHFPLA	KIH68283/337-351	Ancylostoma duodenale	NP_006000	Tubulin alpha-1A	DRB1:15:01					
APWCGHCKALAPEYA	KIH46995/49-63	Ancylostoma duodenale	NP_000909	Disulfide-isomerase	DRB1:09:01					
FMVDNEAIYDICRRN	KIH68283/283-297	Ancylostoma duodenale	NP_006000	Tubulin alpha-1A	DRB3:01:01					
HSDCAFMVDNEAIYD	XP_013298506/193-207	Necator americanus	NP_006000	Tubulin alpha-1A	DRB3:01:01					
LMYAKRAFVHWYVGE	CDW57876/409-423	Trichuris trichiura	NP_006000	Tubulin alpha-1A	DRB1:15:01					
CLLYRGDVVPKDVNA	KIH68283/397-411	Ancylostoma duodenale	NP_006000	Tubulin alpha-1A	DRB3:01:01					
VDLTEFQTNLVPYPR	KIH68283/331-345	Ancylostoma duodenale	NP_006000	Tubulin alpha-1A	NP					
YPAPQVSTAVVEPYN	KIH68283/253-267	Ancylostoma duodenale	NP_006000	Tubulin alpha-1A	NP					
AIAEAWARLDHKFDL	XP_013298506/379-393	Necator americanus	NP_006000	Tubulin alpha-1A	NP					
VNDLPVGRSVDETLR	XP_013307087/601-615	Necator americanus	NP_002565	Peroxiredoxin-1	NP					
SLLMERLSVDYGKKS	CDW57876/163-177	Trichuris trichiura	NP_006000	Tubulin alpha-1A	NP					
SGAGKTENTKKVIQY	XP_013291219/175-189	Necator americanus	NP_002464	Myosin	NP					
GHYTIGKEIIDLVLD	KIH68283/187-201	Ancylostoma duodenale	NP_006000	Tubulin alpha-1A	NP					
YGKKSKLEFSIYPAP	XP_013298506/157-171	Necator americanus	NP_006000	Tubulin alpha-1A	NP					
VSSITASLRFDGALN	CDW57876/247-261	Trichuris trichiura	NP_006000	Tubulin alpha-1A	NP					
DREDQSILCTGESGA	XP_013291219/163-177	Necator americanus	NP_002464	Myosin	NP					
WYVGEGMEEGEFSEA	XP_013298506/403-417	Necator americanus	NP_006000	Tubulin alpha-1A	NP					
HSFGGGTGSGFTSLL	CDW57876/151-165	Trichuris trichiura	NP_006000	Tubulin alpha-1A	NP					
LDIERPTYTNLNRLI	CDW57876/229-243	Trichuris trichiura	NP_006000	Tubulin alpha-1A	NP					
CPTGFKVGINYQPPT	XP_013298506/343-357	Necator americanus	NP_006000	Tubulin alpha-1A	NP					
VLEGIRICRQGFPNR	XP_013291219/697-711	Necator americanus	NP_002464	Myosin	NP					
VGQAGVQIGNACWEL	KIH68283/91-105	Ancylostoma duodenale	NP_006000	Tubulin alpha-1A	NP					
TTHTTLEHSDCAFMV	KIH68283/271-285	Ancylostoma duodenale	NP_006000	Tubulin alpha-1A	NP					
INYQPPTVVPGGDLA	CDW57876/367-381	Trichuris trichiura	NP_006000	Tubulin alpha-1A	NP					
VKNDNSSRFGKFIRI	XP_013291219/235-249	Necator americanus	NP_002464	Myosin	NP					
PAPQVSTAVVEPYNS	XP_013298506/169-183	Necator americanus	NP_006000	Tubulin alpha-1A	NP					
MVDNEAIYDICRRNL	XP_013298506/199-213	Necator americanus	NP_006000	Tubulin alpha-1A	NP					
GGGTGSGFTSLLMER	KIH68283/223-237	Ancylostoma duodenale	NP_006000	Tubulin alpha-1A	NP					
DYGKKSKLEFSIYPA	KIH68283/241-255	Ancylostoma duodenale	NP_006000	Tubulin alpha-1A	NP					
ANNYARGHYTIGKEI	KIH68283/181-195	Ancylostoma duodenale	NP_006000	Tubulin alpha-1A	NP					
EDAANNYARGHYTIG	CDW57876/109-123	Trichuris trichiura	NP_006000	Tubulin alpha-1A	NP					
TGKEDAANNYARGHY	KIH68283/175-189	Ancylostoma duodenale	NP_006000	Tubulin alpha-1A	NP					
VGEGMEEGEFSEARE	CDW57876/421-435	Trichuris trichiura	NP_006000	Tubulin alpha-1A	NP					
SILTTHTTLEHSDCA	CDW57876/199-213	Trichuris trichiura	NP_006000	Tubulin alpha-1A	NP					
GVQIGNACWELYCLE	XP_013298506/13-27	Necator americanus	NP_006000	Tubulin alpha-1A	NP					
DNEAIYDICRRNLDI	CDW57876/217-231	Trichuris trichiura	NP_006000	Tubulin alpha-1A	NP					
MERLSVDYGKKSKLE	KIH68283/235-249	Ancylostoma duodenale	NP_006000	Tubulin alpha-1A	NP					
TEFQTNLVPYPRIHF	CDW57876/265-279	Trichuris trichiura	NP_006000	Tubulin alpha-1A	NP					
FVDWCPTGFKVGINY	CDW57876/355-369	Trichuris trichiura	NP_006000	Tubulin alpha-1A	NP					
IYTYSGLFCVVINPY	XP_013291219/109-123	Necator americanus	NP_002464	Myosin	NP					
QIGNACWELYCLEHG	KIH68283/97-111	Ancylostoma duodenale	NP_006000	Tubulin alpha-1A	NP					
ALNVDLTEFQTNLVP	CDW57876/259-273	Trichuris trichiura	NP_006000	Tubulin alpha-1A	NP					
IHVGQAGVQIGNACW	XP_013298506/7-21	Necator americanus	NP_006000	Tubulin alpha-1A	NP					
IQFVDWCPTGFKVGI	XP_013298506/337-351	Necator americanus	NP_006000	Tubulin alpha-1A	NP					
HPEQLITGKEDAANN	KIH68283-169/183	Ancylostoma duodenale	NP_006000	Tubulin alpha-1A	NP					
THTTLEHSDCAFMVD	XP_013298506/187-201	Necator americanus	NP_006000	Tubulin alpha-1A	NP					
PQVSTAVVEPYNSIL	CDW57876-187/201	Trichuris trichiura	NP_006000	Tubulin alpha-1A	NP					
AEAWARLDHKFDLMY	CDW57876-397/411	Trichuris trichiura	NP_006000	Tubulin alpha-1A	NP					
GKEDAANNYARGHYT	XP_013298506/91-105	Necator americanus	NP_006000	Tubulin alpha-1A	NP					
DLTEFQTNLVPYPRI	XP_013298506/247-261	Necator americanus	NP_006000	Tubulin alpha-1A	NP					
ACWELYCLEHGIQPD	XP_013298506/19-33	Necator americanus	NP_006000	Tubulin alpha-1A	NP					
AFVHWYVGEGMEEGE	CDW57876-415/429	Trichuris trichiura	NP_006000	Tubulin alpha-1A	NP					
MLSNTTAIAEAWARL	XP_013298506/373-387	Necator americanus	NP_006000	Tubulin alpha-1A	NP					
ILCTGESGAGKTENT	XP_013291219/169-183	Necator americanus	NP_002464	Myosin	NP					
GRSVDETLRLVQAFQ	XP_013307087/607-621	Necator americanus	NP_002565	Peroxiredoxin-1	NP					
STAVVEPYNSILTTH	KIH68283/259-273	Ancylostoma duodenale	NP_006000	Tubulin alpha-1A	NP					
DICRRNLDIERPTYT	CDW57876/223-237	Trichuris trichiura	NP_006000	Tubulin alpha-1A	NP					
ERLSVDYGKKSKLEF	XP_013298506/151-165	Necator americanus	NP_006000	Tubulin alpha-1A	NP					
QGASFIGILDIAGFE	XP_013291219/451-465	Necator americanus	NP_002464	Myosin	NP					
TTLEHSDCAFMVDNE	CDW57876/205-219	Trichuris trichiura	NP_006000	Tubulin alpha-1A	NP					

Key to table columns:
Peptide sequence: Amino acid sequence of peptides in ES-antigens from common human intestinal nematodes (hIN) with 100% identity over the entire length to human self antigens (all peptides have 15 amino acid residues).
ES antigen: Accession number of hIN ES antigen, followed by the position of the peptide in the amino acid sequence of the antigen
hIN species: Source of nematode species
Human antigen: Accession number of human antigen
Antigen name: Name of human antigen
Predicted HLA II binding profile: HLA-DR molecules with the specified HLA-DRB chains that were predicted to bind the noted peptides. NP: None Predicted
