# Supplementary material for: Peptides Derived from α-Tubulin Induce Functional T Regulatory Cells
Source: Int J Mol Sci. 2025 Aug 28;26(17):8356. doi: 10.3390/ijms26178356 (PMC12542834; doi:10.3390/ijms26178356)
Supplement: Supplementary file 1 [file ijms-26-08356-s001.zip › Supplementary_Table S2.pdf]

Supplementary Table S2: Conservation of potential Treg cell epitopes in different α-tubulin isoforms

Peptide sequence	TUBA1A	TUBA1B	TUBA1C	TUBA3C	TUBA3D	TUBA3E	TUBA4A	TUBA4B	TUBA8	TUBAL3
LDHKFDLMYAKRAFV	LDHKFDLMYAKRAFV (100.00)	LDHKFDLMYAKRAFV (100.00)	LDHKFDLMYAKRAFV (100.00)	LDHKFDLMYAKRAFV (100.00)	LDHKFDLMYAKRAFV (100.00)	LVHKFDLMYAKWAFV (86.67)	LDHKFDLMYAKRAFV (100.00)	–	LDHKFDLMYAKRAFV (100.00)	LDHKFDLMYAKRAFL (93.33)
FDLMYAKRAFVHWYV	FDLMYAKRAFVHWYV (100.00)	FDLMYAKRAFVHWYV (100.00)	FDLMYAKRAFVHWYV (100.00)	FDLMYAKRAFVHWYV (100.00)	FDLMYAKRAFVHWYV (100.00)	FDLMYAKWAFVHWYV (93.33)	FDLMYAKRAFVHWYV (100.00)	–	FDLMYAKRAFVHWYV (100.00)	FDLMYAKRAFLHWYL (86.67)
RLIGQIVSSITASLR	RLIGQIVSSITASLR (100)	RLISQIVSSITASLR (93.33)	RLISQIVSSITASLR (93.33)	RLIGQIVSSITASLR (100.00)	RLIGQIVSSITASLR (100.00)	RLIGQIVSSITASLR (100.00)	RLISQIVSSITASLR (93.33)	RLISQIVSSITASLR (93.33)	RLISQIVSSITASLR (93.33)	RLVVQVVSSITASLR (80.00)
ITASLRFDGALNVDL	ITASLRFDGALNVDL (100.00)	ITASLRFDGALNVDL (100.00)	ITASLRFDGALNVDL (100.00)	ITASLRFDGALNVDL (100.00)	ITASLRFDGALNVDL (100.00)	ITASLRFDGALNVDL (100.00)	ITASLRFDGALNVDL (100.00)	ITASLRFDGALNVDL (100.00)	ITASLRFDGALNVDL (100.00)	ITASLRFEGPLNVDL (86.67)
RAVCMLSNTTAIAEA	RAVCMLSNTTAIAEA (100.00)	RAVCMLSNTTAIAEA (100.00)	RAVCMLSNTTAVAEA (93.33)	RAVCMLSNTTAIAEA (100.00)	RAVCMLSNTTAIAEA (100.00)	RAVCMLSNTTAIAEA (100.00)	RAVCMLSNTTAIAEA (100.00)	–	RAVCMLSNTTAIAEA (100.00)	RSICMLSNTTAIVEA (80.00)
PYNSILTTHTTLEHS	PYNSILTTHTTLEHS (100.00)	PYNSILTTHTTLEHS (100.00)	PYNSILTTHTTLEHS (100.00)	PYNSILTTHTTLEHS (100.00)	PYNSILTTHTTLEHS (100.00)	PYNSILTTHTTLEHS (100.00)	PYNSILTTHTTLEHS (100.00)	PYNSILTTHTTLEHS (100.00)	PYNSILTTHTTLEHS (100.00)	PYNSVLTTHSTTEHT (73.33)
NLNRLIGQIVSSITA	NLNRLIGQIVSSITA (100.00)	NLNRLISQIVSSITA (93.00)	NLNRLISQIVSSITA (100.00)	NLNRLIGQIVSSITA (93.33)	NLNRLIGQIVSSITA (93.33)	NLNRLIGQIVSSITA (93.33)	NLNRLISQIVSSITA (100.00)	NLNRLISQIVSSITA (100.00)	NLNRLISQIVSSITA (100.00)	SINRLVVQVVSSITA (66.67)
GGTGSGFTSLLMERL	GGTGSGFTSLLMERL (100.00)	GGTGSGFTSLLMERL (100.00)	GGTGSGFTSLLMERL (100.00)	FASLLMERL (88.89)	FASLLMERL (88.89)	FASLLMERL (88.89)	GGTGSGFTSLLMERL (100.00)	GTGSDVTSFLMEWL (71.43)	GGTGSGFTSLLMERL (100.00)	GGTGSGFTSLLMERL (100.00)
ARLDHKFDLMYAKRA	ARLDHKFDLMYAKRA (100.00)	ARLDHKFDLMYAKRA (100.00)	ARLDHKFDLMYAKRA (100.00)	ARLDHKFDLMYAKRA (100.00)	ARLDHKFDLMYAKRA (100.00)	ARLVHKFDLMYAKWA (86.67)	ARLDHKFDLMYAKRA (100.00)	–	ARLDHKFDLMYAKRA (100.00)	ARLDHKFDLMYAKRA (100.00)
VVEPYNSILTTHTTL	VVEPYNSILTTHTTL (100.00)	VVEPYNSILTTHTTL (100.00)	VVEPYNSILTTHTTL (100.00)	VVEPYNSILTTHTTL (100.00)	VVEPYNSILTTHTTL (100.00)	VVEPYNSILTTHTTL (100.00)	VVEPYNSILTTHTTL (100.00)	MVQPYNSILTTHTTL (86.67)	VVEPYNSILTTHTTL (100.00)	VVEPYNSVLTTHST (85.71)
TGSGFTSLLMERLSV	TGSGFTSLLMERLSV (100.00)	TGSGFTSLLMERLSV (100.00)	TGSGFTSLLMERLSV (100.00)	SLLMERLSV (100.00)	SLLMERLSV (100.00)	SLLMERLSV (100.00)	TGSGFTSLLMERLSV (100.00)	TGSDVTSFLMEWLSV (73.33)	SLLMERLSL (88.89)	SLLMERLT (87.50)
GFTSLLMERLSVDYG	GFTSLLMERLSVDYG (100.00)	GFTSLLMERLSVDYG (100.00)	GFTSLLMERLSVDYG (100.00)	SLLMERLSVDYG (100.00)	SLLMERLSVDYG (100.00)	SLLMERLSVDY (100.00)	GFTSLLMERLSVDYG (100.00)	TSFLMEWLSVNYG (76.92)	LLMERLSLDYG (90.91)	LLMERLTGEY (70.00)
TAVVEPYNSILTTHT	TAVVEPYNSILTTHT (100.00)	TAVVEPYNSILTTHT (100.00)	TAVVEPYNSILTTHT (100.00)	TAVVEPYNSILTTHT (100.00)	TAVVEPYNSILTTHT (100.00)	TAVVEPYNSILTTHT (100.00)	TAVVEPYNSILTTHT (100.00)	TAMVQPYNSILTTHT (86.67)	TAVVEPYNSILTTHT (100.00)	TAVVEPYNSVLTTHS (86.67)
SLRFDGALNVDLTEF	SLRFDGALNVDLTEF (100.00)	SLRFDGALNVDLTEF (100.00)	SLRFDGALNVDLTEF (100.00)	SLRFDGALNVDLTEF (100.00)	SLRFDGALNVDLTEF (100.00)	SLRFDGALNVDLTEF (100.00)	SLRFDGALNVDLTEF (100.00)	SLRFDGALNVDLTEF (100.00)	SLRFDGALNVDLTEF (100.00)	SLRFEGPLNVDLIEF (80.00)
YNSILTTHTTLEHSD	YNSILTTHTTLEHSD (100.00)	YNSILTTHTTLEHSD (100.00)	YNSILTTHTTLEHSD (100.00)	YNSILTTHTTLEHSD (100.00)	YNSILTTHTTLEHSD (100.00)	YNSILTTHTTLEHSD (100.00)	YNSILTTHTTLEHSD (100.00)	YNSILTTHTTLEHSD (100.00)	YNSILTTHTTLEHSD (100.00)	YNSVLTTHSTTEHTD (73.33)
TGFKVGINYQPPTVV	TGFKVGINYQPPTVV (100.00)	TGFKVGINYQPPTVV (100.00)	TGFKVGINYQPPTVV (100.00)	TGFKVGINYQPPTVV (100.00)	TGFKVGINYQPPTVV (100.00)	TGFKVGINYQPPTVV (100.00)	TGFKVGINYQPPTVV (100.00)	–	TGFKVGINYQPPTVV (100.00)	TGFKVGINNRPPTVM (80.00)
DCAFMVDNEAIYDIC	DCAFMVDNEAIYDIC (100.00)	DCAFMVDNEAIYDIC (100.00)	DCAFMVDNEAIYDIC (100.00)	DCAFMVDNEAIYDIC (100.00)	DCAFMVDNEAIYDIC (100.00)	DCAFMVDNEAIYDIC (100.00)	DCAFMVDNEAIYDIC (100.00)	DCAFMVDNKAIYDIC (93.33)	DCAFMVDNEAIYDIC (100.00)	DCTFMVDNEAVYDIC (86.67)
LEFSIYPAPQVSTAV	LEFSIYPAPQVSTAV (100.00)	LEFSIYPAPQVSTAV (100.00)	LEFSIYPAPQVSTAV (100.00)	LEFAIYPAPQVSTAV (93.33)	LEFAIYPAPQVSTAV (93.33)	LEFAIYPAPQVSTAV (93.33)	LEFSIYPAPQVSTAV (100.00)	LGFSIYPAPQVSTAM (86.67)	LEFAIYPAPQVSTAV (93.33)	LEFSVYPAPRISTAV (80.00)
KLEFSIYPAPQVSTA	KLEFSIYPAPQVSTA (100.00)	KLEFSIYPAPQVSTA (100.00)	KLEFSIYPAPQVSTA (100.00)	KLEFAIYPAPQVSTA (93.33)	KLEFAIYPAPQVSTA (93.33)	KLEFAIYPAPQVSTA (93.33)	KLEFSIYPAPQVSTA (100.00)	KLGFSIYPAPQVSTA (93.33)	KLEFAIYPAPQVSTA (93.33)	KLEFSVYPAPRISTA (80.00)
RTGTYRQLFHPEQLI	RTGTYRQLFHPEQLI (100.00)	RTGTYRQLFHPEQLI (100.00)	RTGTYRQLFHPEQLI (100.00)	RTGTYRQLFHPEQLI (100.00)	RTGTYRQLFHPEQLI (100.00)	RTGTYRQLFHPEQLI (100.00)	RNGPYRQLFHPEQLI (86.67)	GTYRQIFHPEQLI (92.31)	RAGTYRQLFHPEQLI (93.33)	RTGQHRSLFHPEQLL (73.33)
FTSLLMERLSVDYGK	FTSLLMERLSVDYGK (100.00)	FTSLLMERLSVDYGK (100.00)	FTSLLMERLSVDYGK (100.00)	SLLMERLSVDYGK (100.00)	SLLMERLSVDYGK (100.00)	SLLMERLSVDYSK (92.31)	FTSLLMERLSVDYGK (100.00)	TSFLMEWLSVNYGK (78.57)	LLMERLSLDYGK (91.67)	LLMERLTGEYSR (58.33)
YRGDVVPKDVNAAIA	YRGDVVPKDVNAAIA (100.00)	YRGDVVPKDVNAAIA (100.00)	YRGDVVPKDVNAAIA (100.00)	YRGDVVPKDVNAAIA (100.00)	YRGDVVPKDVNAAIA (100.00)	YRGDVVPKDVNAAIA (100.00)	YRGDVVPKDVNAAIA (100.00)	–	YRGDVVPKDVNVAIA (93.33)	YRGDVVPKEVNAAIA (93.33)
EHSDCAFMVDNEAIY	EHSDCAFMVDNEAIY (100.00)	EHSDCAFMVDNEAIY (100.00)	EHSDCAFMVDNEAIY (100.00)	EHSDCAFMVDNEAIY (100.00)	EHSDCAFMVDNEAIY (100.00)	EHSDCAFMVDNEAIY (100.00)	EHSDCAFMVDNEAIY (100.00)	EHSDCAFMVDNKAIY (93.33)	EHSDCAFMVDNEAIY (100.00)	EHTDCTFMVDNEAVY (80.00)
SNTTAIAEAWARLDH	SNTTAIAEAWARLDH (100.00)	SNTTAIAEAWARLDH (100.00)	SNTTAVAEAWARLDH (93.33)	SNTTAIAEAWARLDH (100.00)	SNTTAIAEAWARLDH (100.00)	SNTTAIAEAWARLVH (93.33)	SNTTAIAEAWARLDH (100.00)	–	SNTTAIAEAWARLDH (100.00)	SNTTAIVEAWARLDH (93.33)
VGINYQPPTVVPGGD	VGINYQPPTVVPGGD (100.00)	VGINYQPPTVVPGGD (100.00)	VGINYQPPTVVPGGD (100.00)	VGINYQPPTVVPGGD (100.00)	VGINYQPPTVVPGGD (100.00)	VGINYQPPTVVPGGD (100.00)	VGINYQPPTVVPGGD (100.00)	–	VGINYQPPTVVPGGD (100.00)	VGINNRPPTVMPGGD (80.00)
FDGALNVDLTEFQTN	FDGALNVDLTEFQTN (100.00)	FDGALNVDLTEFQTN (100.00)	FDGALNVDLTEFQTN (100.00)	FDGALNVDLTEFQTN (100.00)	FDGALNVDLTEFQTN (100.00)	FDGALNVDLTEFQTN (100.00)	FDGALNVDLTEFQTN (100.00)	FDGALNVDLTEFQTN (100.00)	FDGALNVDLTEFQTN (100.00)	FEGPLNVDLIEFQTN (80.00)
TNLVPYPRIHFPLAT	TNLVPYPRIHFPLAT (100.00)	TNLVPYPRIHFPLAT (100.00)	TNLVPYPRIHFPLAT (100.00)	TNLVPYPRIHFPLAT (100.00)	TNLVPYPRIHFPLAT (100.00)	TNLVPYPRIHFPLAT (100.00)	TNLVPYPRIHFPLAT (100.00)	–	TNLVPYPRIHFPLVT (93.33)	TNLVPYPRIHFPM (92.31)
KRAFVHWYVGEGMEE	KRAFVHWYVGEGMEE (100.00)	KRAFVHWYVGEGMEE (100.00)	KRAFVHWYVGEGMEE (100.00)	KRAFVHWYVGEGMEE (100.00)	KRAFVHWYVGEGMEE (100.00)	KWAFVHWYVGEGMEE (93.33)	KRAFVHWYVGEGMEE (100.00)	–	KRAFVHWYVGEGMEE (100.00)	KRAFLHWYLREGMEE (80.00)
TYAPVISAEKAYHEQ	TYAPVISAEKAYHEQ (100.00)	TYAPVISAEKAYHEQ (100.00)	TYAPVISAEKAYHEQ (100.00)	TYAPVISAEKAYHEQ (100.00)	TYAPVISAEKAYHEQ (100.00)	TYAPVISAEKAYHEQ (100.00)	TYAPVISAEKAYHEQ (100.00)	–	TYAPIISAEKAYHEQ (93.33)	FAPIVSADKAYHEQ (71.43)
QTNLVPYPRIHFPLA	QTNLVPYPRIHFPLA (100.00)	QTNLVPYPRIHFPLA (100.00)	QTNLVPYPRIHFPLA (100.00)	QTNLVPYPRIHFPLA (100.00)	QTNLVPYPRIHFPLA (100.00)	QTNLVPYPRIHFPLA (100.00)	QTNLVPYPRIHFPLA (100.00)	–	QTNLVPYPRIHFPL (100.00)	QTNLVPYPRIHFPM (92.86)
FMVDNEAIYDICRRN	FMVDNEAIYDICRRN (100.00)	FMVDNEAIYDICRRN (100.00)	FMVDNEAIYDICRRN (100.00)	FMVDNEAIYDICRRN (100.00)	FMVDNEAIYDICRRN (100.00)	FMVDNEAIYDICRRN (100.00)	FMVDNEAIYDICRRN (100.00)	FMVDNKAIYDICHCN (80.00)	FMVDNEAIYDICRRN (100.00)	FMVDNEAVYDICHR (85.71)
HSDCAFMVDNEAIYD	HSDCAFMVDNEAIYD (100.00)	HSDCAFMVDNEAIYD (100.00)	HSDCAFMVDNEAIYD (100.00)	HSDCAFMVDNEAIYD (100.00)	HSDCAFMVDNEAIYD (100.00)	HSDCAFMVDNEAIYD (100.00)	HSDCAFMVDNEAIYD (100.00)	HSDCAFMVDNKAIYD (93.33)	HSDCAFMVDNEAIYD (100.00)	HTDCTFMVDNEAVYD (80.00)
LMYAKRAFVHWYVGE	LMYAKRAFVHWYVGE (100.00)	LMYAKRAFVHWYVGE (100.00)	LMYAKRAFVHWYVGE (100.00)	LMYAKRAFVHWYVGE (100.00)	LMYAKRAFVHWYVGE (100.00)	LMYAKWAFVHWYVGE (93.33)	LMYAKRAFVHWYVGE (100.00)	–	LMYAKRAFVHWYVGE (100.00)	LMYAKRAFLHWYLRE (80.00)
CLLYRGDVVPKDVNA	CLLYRGDVVPKDVNA (100.00)	CLLYRGDVVPKDVNA (100.00)	CLLYRGDVVPKDVNA (100.00)	CMLYRGDVVPKDVNA (93.33)	CMLYRGDVVPKDVNA (93.33)	CMLYRGDVVPKDVNA (93.33)	CLLYRGDVVPKDVNA (100.00)	–	CMLYRGDVVPKDVN (92.86)	CLLYRGDVVPKEVNA (93.33)

The columns represent the gene codes of different α-tubulin isoforms and equivalent peptide sequences between them corresponding to potential Treg cell epitopes are identified and identity reported in percentage in brackets. A dash (-) indicates that the peptide was not found in that isoform.
